# Non‐linear models of species' responses to environmental and spatial gradients

**DOI:** 10.1111/ele.14121

**Published:** 2022-10-21

**Authors:** Marti J. Anderson, Daniel C. I. Walsh, Winston L. Sweatman, Andrew J. Punnett

**Affiliations:** ^1^ New Zealand Institute for Advanced Study (NZIAS) Massey University Auckland New Zealand; ^2^ PRIMER‐e (Quest Research Limited) Auckland New Zealand; ^3^ Statistical Consulting Centre University of Auckland Auckland New Zealand; ^4^ School of Mathematical and Computational Sciences Massey University Auckland New Zealand

**Keywords:** abundance, biomass, counts, cover, ecological statistics, environmental variables, gradient analysis, latitude, species distribution models, zero‐inflation

## Abstract

Species' responses to broad‐scale environmental or spatial gradients are typically unimodal. Current models of species' responses along gradients tend to be overly simplistic (e.g., linear, quadratic or Gaussian GLMs), or are suitably flexible (e.g., splines, GAMs) but lack direct ecologically interpretable parameters. We describe a parametric framework for species‐environment non‐linear modelling (‘*senlm*’). The framework has two components: (i) a non‐linear parametric mathematical function to model the mean species response along a gradient that allows asymmetry, flattening/peakedness or bimodality; and (ii) a statistical error distribution tailored for ecological data types, allowing intrinsic mean–variance relationships and zero‐inflation. We demonstrate the utility of this model framework, highlighting the flexibility of a range of possible mean functions and a broad range of potential error distributions, in analyses of fish species' abundances along a depth gradient, and how they change over time and at different latitudes.

## Graphical abstract

We describe a new framework for non‐linear models of species to environmental or spatial gradients, along with an associated R package, 'senlm'. The framework has two essential components: (i) a non‐linear parametric mathematical function to model the mean species response curve along the gradient that allows for asymmetry, flattening/peakedness or bimodality; and (ii) a statistical error distribution tailored for ecological data types (counts, densities, biomass, cover, presence/absence, etc.), that allows for intrinsic mean‐variance relationships and zero‐inflation. The utility of the framework is demonstrated in analyses of fish abundances along a depth gradient, also showing how their responses can change over time and at different latitudes.

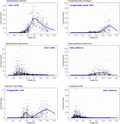

## INTRODUCTION

Gradient analysis has a long history in ecology, reaching back to Whittaker's seminal work (1956, 1967). Responses of species along broad‐scale spatial gradients (such as latitude, depth or elevation), or environmental gradients (such as nutrients, light, temperature or moisture) are typically unimodal (Gauch et al., 1974; Jamil & ter Braak, 2013; ter Braak, 1996; Westman, 1980; Whittaker, 1956, 1967). Each species is thought to have an ‘optimum’ (modal) value along a given gradient (e.g., the temperature which best suits the species' survival—neither too cold nor too hot, but ‘just right’). Different species are expected to have different optimal positions and different ‘tolerances’ (spread) along the gradient, according to their realised niche (Colwell & Rangel, 2009; Whittaker et al., 1973). Multiple species typically show a pattern of (partially) overlapping unimodal curves along a gradient of interest (ter Braak, 1996; Whittaker, 1956, 1967).

Unimodal responses of species to environmental gradients have historically been modelled using a bell‐shaped or Gaussian curve (Gauch et al., 1974; Jamil & ter Braak, 2013; Johnson & Goodall, 1980; ter Braak, 1985, 1986, 1996; Westman, 1980; Yee, 2004). A generalised linear model (GLM) that includes a quadratic term might also be used (Austin et al., 1984; Makarenkov & Legendre, 2002; ter Braak & Looman, 1986; Warton et al., 2015). Although the physiological response of a species might be approximately bell‐shaped, other factors, such as biogeographical history, unmeasured environmental variables, dispersal limitation, sampling extent, predation, herbivory or competition can all substantially alter this shape (Austin, 1976, 1980; Bradshaw et al., 2014; Oksanen & Minchin, 2002). Empirically, mean species' responses show a wide variety of shapes; they can be asymmetric, j‐shaped, truncated, peaked, flattened, plateaued, bimodal or even multi‐modal. Clearly, a broader range of non‐linear mathematical forms should be explored (Oksanen & Minchin, 2002).

Two logistic curves can be combined to create a suitable shape for the mean unimodal response that allows for both asymmetry and peakedness/flattening (Huisman et al., 1993; Jansen & Oksanen, 2013), but these have thus far only been applied to presence/absence or cover data with an upper bound. Generalised additive models (GAMs, Hastie & Tibshirani, 1990; Yee, 2015), including B‐splines (de Boor, 2001) and P‐splines (Eilers & Marx, 2021), are more flexible, and can take virtually any shape (Anderson, 2008; Fraaije et al., 2015; Rigby & Stasinopoulos, 2005; Yee & Mitchell, 1991). However, they can over‐fit (so need some form of cross‐validation, Gu & Wahba, 1991), may be adversely affected by excess zeros and do not yield directly interpretable parameters. More sophisticated predictive models, such as artificial neural networks (Harrison et al., 2006; Lek & Guégan, 1999), maximum entropy models (Phillips et al., 2006), regression trees/splines (Chipman et al., 2010; Elith et al., 2008; Leathwick et al., 2005; Stoklosa & Warton, 2018), or ensemble models (Araújo & New, 2007) also lack directly interpretable parameters, and many deal only with presence/absence data (Elith & Leathwick, 2009).

Useful parameters for characterising a species' mean response include the modal position, *m*, of the species along the gradient (directly interpretable as its optimum), and the mean abundance of the species at that modal position, that is, the maximum height, *H*, of the non‐linear response curve. Armed with estimates of such parameters for a given species, one may then track changes in their values over time or across space. For example, a warm‐water species' modal position along a latitudinal gradient may shift towards the poles due to climate change, as temperatures increase at higher latitudes over time.

The mean response is, however, just part of the story. Real field data are quite messy (Legendre, 1993). Counts of individuals (or density or biomass) have no upper bound, and typically display large residual variance (Chapman & Underwood, 2008), zero‐inflation (Martin et al., 2005; Smith et al., 2012), over‐dispersion and intrinsic variance–mean relationships (McArdle et al., 1990; McArdle & Anderson, 2004; Taylor, 1961).

Here, we describe a modular approach for modelling the non‐linear response of a given species along an environmental or spatial gradient of interest, termed ‘*senlm*’ (for species‐environment non‐linear models). This model framework couples together two key elements: (i) a flexible, parametric, non‐linear mathematical function for the mean response; and (ii) a statistical error distribution to model residual variation around the mean that will accommodate noisy and generally zero‐inflated ecological field data. We focus here on a suite of statistical error distributions appropriate for the types of data used to quantify the abundance of organisms in the field (i.e., counts, biomass, densities, frequencies, cover or presence/absence). We demonstrate the utility of our proposed model framework for uncovering novel insights on species' distributions along gradients *via* analyses of North‐east Pacific groundfish species *versus* depth.

## DESCRIPTION OF THE METHOD

### General modelling framework

Let Y be a non‐negative random variable with observed values $y_{i}$ (counts, densities, biomass, percentage cover, or presence/absence, etc.) quantifying the abundance (or relative abundance, biomass or occurrence) of a single species obtained from i=1,…,N standardised sampling units situated at positions $x_{i}$ along gradient X of interest. For presence/absence or percentage cover, values are bounded inclusively between 0 and 1 (or 0 and 100%). Our general model considers $y_{i}$ to be drawn independently from probability distribution $P\left(\mu_{i},\theta_{E}\right)$, where:

- $\mu_{i}$ is the parameter specifying the mean of P, defined by a non‐linear function $f\left(x;\theta_{M}\right)$ of x, requiring a set of parameters $\theta_{M}$; so $\mu_{i}=f\left(x_{i};\theta_{M}\right)$ at position $x_{i}$; and
- $\theta_{E}$ is the set of all other required parameters of P excluding the mean.

For example, if P is a zero‐inflated negative binomial (ZINB), $\theta_{E}=\left\{\phi ,\pi \right\}$, with ϕ being the dispersion parameter and π the probability of an excess zero, then the model is $y_{i}\sim \text{ZINB}\left(\mu_{i},\phi ,\pi \right)$, with $\mu_{i}=f\left(x_{i};\theta_{M}\right)$.

### The mean response

The mean response function first must be sufficient to characterise a classic unimodal, bell‐shaped form, as predicted by theory, with a special focus on estimating the maximum height (*H*) of the curve (i.e., its mode), particularly for data types (abundance, density, biomass) that have no known upper bound, and also the modal position (*m*) along the gradient at which that maximum height occurs (i.e., the species' ‘optimum’). The mean function must also allow for: (i) asymmetry, either to the right or the left; for example, a species may display a more rapid decrease in mean abundance if its tolerance/adaptability to non‐optimal conditions is exceeded more quickly in one direction (e.g., too hot) *versus* the other (e.g., too cold); (ii) flattened or pointed/peaked shapes (e.g., a species may require highly specific environmental conditions, or it may be very broadly distributed); (iii) j‐shaped, to either the left or right (e.g., due to the sampling window covering only part of a species' range); and (iv) potential bi‐ or multi‐modal mean responses, with each mode being potentially of different heights (e.g., a species may encounter heavy competition or may be limited by some other environmental parameter at its physiological optimum position along a measured gradient, hence show a ‘dip’ in its response, with peaks either side of this).

Mean response functions in our *senlm* framework (Table 1) include two new mathematical functions that allow a suitably flexible variety of shapes typical of species' responses to gradients: a modified *beta* function (following Austin, 1976), and a modified *sech* function. We highlight here, in particular, the modified *sech* function, designed specifically to provide ecologically relevant estimates of parameters m and H for a species' mean response directly.

**TABLE 1:** Mean functions and error distributions currently included in the *senlm* framework for modelling non‐linear species' responses to environmental or spatial gradients. Any mean function (column 1) can be paired with any error distribution (column 2) for a given species. Acronyms identify zero‐inflated error distributions that have a probability of an excess zero that is constant (‘ZI…’), linked to the log of the mean (‘ZI…L’) or linked directly to the mean itself (‘ZI…L.mu’) along the gradient

Mean functions	Error distributions
Constant	*Discrete (counts; abundances)*
Uniform	Poisson
Gaussian	Zero‐inflated Poisson (ZIP; ZIPL; ZIPL.mu)
HOF	Negative binomial (NB)
Beta	Zero‐inflated negative binomial (ZINB; ZINBL; ZINBL.mu)
Sech	*Continuous (biomass; densities; traits)*
Mixed Gaussian	Gamma
	Zero‐inflated gamma (ZIG; ZIGL; ZIGL.mu)
	Zero‐inflated inverse Gaussian (ZIIG; ZIIGL; ZIIGL.mu)
	Tweedie
	*Cover (percentages or proportions)*
	Binomial
	Tail‐adjusted beta (TAB)
	Zero‐inflated tail‐adjusted beta (ZITAB)
	*Binary (presence/absence)*
	Bernoulli

A basic *sech* function has the following form: $\mathrm{sech}\left(x\right)=2/\left(e^{x}+e^{-x}\right)$. This is a symmetric, unimodal function centred on zero. Our new proposed function has parameters $\theta_{M}=\left\{H,m,s,r,p\right\}$ and is given by the following equation:

$$f_{\mathrm{sech}}\left(x;\theta_{M}\right)=\frac{H}{H_{m}}\exp\left(\frac{\mathrm{rp}}{s}\left(x-\left(m-x_{m}\right)\right)\right){\left(\mathrm{sech}\left(\frac{x-\left(m-x_{m}\right)}{s}\right)\right)}^{p}$$

where:

H = Maximum value of the mean function $\left(H>0.\right)$

m = Location of the maximum $\left(-\infty <m<\infty \right)$

s= Spread parameter $\left(s>0\right)$

r = Symmetry parameter $\left(-1<r<1\right)$

p = Peakedness parameter $\left(p>0\right)$

The values $x_{m}=\frac{s}{2}\log\left(\frac{1+r}{1-r}\right)$ and $H_{m}=\exp\left(\frac{\mathrm{rp}}{s}x_{m}\right)$ ${\left(\sqrt{1-r^{2}}\right)}^{p}$ are used to fix the curve so that m is the location of the mode and H is the maximum height of the curve (at m). If r is positive (or negative) the curve is asymmetric with a broader right‐hand (or left‐hand) tail. Note that a simpler model (with fewer parameters) may be obtained by setting fixed values *a priori* for peakedness (p=1) and/or symmetry (r=0).

We also extended and re‐parameterised mixed logistic functions (called *HOF* models after Huisman et al., 1993; see also Oksanen & Minchin, 2002; Jansen & Oksanen, 2013) so they may accommodate unbounded and zero‐inflated data types. Classic Gaussian mean response curves (Gauch et al., 1974; Jamil & ter Braak, 2013; ter Braak, 1985, 1986, 1996; Westman, 1980; Yee, 2004) are also included within our framework. In addition, we propose the use of mixture models to accommodate bi‐modal or multi‐modal distributions; the Gaussian mixture is an obvious choice here, but mixtures of *sech*, *HOF* and/or *beta* functions (allowing asymmetry, flattened/peaked shapes for any given mode, etc.) might also be considered.

An intuitive understanding of these mean functions may be obtained by referring to the ‘Articles’ page of the *senlm* package on GitHub (https://primer‐e.github.io/senlm/articles/), where visual interactive tools demonstrating each function are provided; specifically, one can move a slider for any parameter in any given function and witness its effect on the shape of the resulting species' non‐linear mean response curve along the gradient.

### The error distribution

Species' responses typically are measured from standardised sampling units as non‐negative values. We can classify and characterise typical data types used to quantify species abundances or occurrences and, for each of these, we propose appropriate probabilistic error distributions, P, as follows (Table 1):

- *count data* (abundances)—discrete, non‐negative, no upper bound, typically over‐dispersed, and with variance–mean relationships; P is Poisson (P), negative binomial (NB) or zero‐inflated versions of these (ZIP or ZINB).
- *biomass data* or *densities*—like counts, but continuous rather than discrete; P is Gamma (G), zero‐inflated Gamma (ZIG), inverse Gaussian (IG), zero‐inflated inverse Gaussian (ZIIG) or Tweedie (T).
- *percentage cover* (or *proportional*) data—continuous with a lower bound at zero and an upper bound of 100% (or 1.0); P is binomial (Bin), or a tail‐adjusted Beta (TAB) with rounding parameter δ. The TAB distribution is identical to a standard Beta distribution between δ and 1−δ, but the probability density below δ is distributed uniformly over the region $\left[0,\left.\delta \right)\right.$ and the probability density above 1−δ is distributed uniformly over the region $\left(1-\delta \right.,\left.1\right]$. This tail‐adjustment allows the distribution to model data that range from 0 to 1 (inclusive). A zero‐inflated version of this is ZITAB.
- *presence/absence* data—binary (0,1); P is Bernoulli.

#### Zero‐inflation

We wish to cater explicitly for potential zero‐inflation, regardless of the data type. Considering responses along a single gradient (X), we expect that a species might well be absent from many samples, even at its optimal position (m) along X, due to variation in the species' response across a host of other unmeasured environmental or biological parameters (e.g., Anderson, 2008). For zero‐inflated (ZI) models, the probability of an excess zero (π) is an additional parameter in $\theta_{E}$. For example, if the error distribution were (say) ZINB, then we would have:

$$P\left(Y_{i}=y_{i}\;|\;\theta_{M},\theta_{E},x_{i}\right)\sim \text{ZINB}\left(\mu_{i},\pi ,\phi \right)$$

$$\;\sim \left\{\begin{array}{c} \mathrm{NB}\left(\mu_{i},\phi \right)\;\text{with probability}\;\left(1-\pi \right)\\ 0\;\text{with probability}\;\pi \end{array}\right.$$

Note that for all zero‐inflated models in this *senlm* framework, we shall use $\mu_{i}$ to denote the mean of that portion of the specified error distribution that does *not* include excess zeros. In the above example, $\mu_{i}$ is the mean of the NB distribution within the ZINB mixture. For error distributions that do not have excess zeros (implicitly, π=0), the expected value of the response variable is $E\left(Y_{i}\right)=\mu_{i}$. However, for zero‐inflated models, $E\left(Y_{i}\right)=\left(1-\pi \right)\mu_{i}$. Although here we shall maintain our focus on $\mu_{i}$, one may clearly choose to focus instead on $E\left(Y_{i}\right)$ in different inferential contexts.

#### Linking the probability of an excess zero to the mean

We acknowledge the well‐known occupancy–abundance relationship in ecology (e.g., Borregaard & Rahbek, 2010; Brown, 1984). Consequently, we can expect more zeros to occur where mean abundances are low. Thus, the degree of zero‐inflation generally is expected to increase with decreases in mean abundance (Nielsen et al., 2005; Sileshi et al., 2009; Smith et al., 2012). To accommodate this parsimoniously, one may allow π to vary along the gradient by linking it directly to the mean (Lambert, 1992; Smith et al., 2012): that is, $\log\left(\frac{\pi_{i}}{1-\pi_{i}}\right)=\gamma_{0}+\gamma_{1}\log\left(\mu_{i}\right)$, resulting in *linked zero‐inflated models*. Note that we expect the parameter, $\gamma_{1}$, to be negative.

We shall denote these linked models by adding an ‘L’ (for ‘linked’) to each of the potential zero‐inflated distributions considered here: *viz*. ZIPL, ZINBL, ZIGL or ZIIGL. In some cases, a better‐fitting model may be achieved by linking the zero‐inflation parameter to the mean directly, rather than to the log of the mean; that is, $\log\left(\frac{\pi_{i}}{1-\pi_{i}}\right)=\gamma_{0}+\gamma_{1}\mu_{i}$. Such models are denoted here by adding ‘.mu’ to the acronym (e.g., ZINBL.mu). One might also consider allowing dispersion parameters to vary along the gradient (e.g., McArdle & Anderson, 2004), but this refinement is not pursued here. Further details and mathematical descriptions of these error distributions are given in *senlm* R package documentation (see ‘Implementation’ below).

## IMPLEMENTATION

We provide an R package, *senlm* (‘species‐environment non‐linear models’), available at the following GitHub repository: https://primer‐e.github.io/senlm/, to implement the model framework. The end‐user can: (i) fit any one or more chosen mean functions with any one or more chosen error distributions to a given set of data (Y,X), in any (or all possible) combinations; (ii) estimate parameters using maximum likelihood (ML) and calculate information criteria (e.g., AIC, AICc or BIC) to aid in choosing among competing potential models; and (iii) produce graphics to visualise species' responses along gradients.

For example, once the *senlm* package has been installed and loaded (library(senlm)), along with data (e.g., suppose one has a data frame called ‘my.data’ containing a gradient variable called ‘env.gradient’ and a response variable called ‘my.species’), then fitting a given model using the senlm() function is straightforward; the user simply provides the data and identifies the mean function and the error distribution desired for the model fit, as follows:
 fit <‐ senlm(data = my.data, xvar = "env.gradient", yvar = "my.species",
 mean_fun = "sech", err_dist = "zinbl")



Standard errors on ML parameter estimates may be obtained in the usual way *via* Fisher's information matrix (McCullagh & Nelder, 1983; McCulloch & Searle, 2001) or *via* a jack‐knife or bootstrap (Chernick, 2008; Davison & Hinkley, 1997). Although we stayed within a classical ML framework here, a Bayesian implementation (Gelman et al., 2013), with specification of suitable priors on parameters, is also possible (e.g., McElreath, 2020). We provide here a vignette with example code (Rcode_S1.txt) and data (Data_S1.csv) to demonstrate use of the *senlm* R package (see ‘Vignette_S1.pdf’ in Supporting Information).

We chose to create a tailored *senlm* R package to focus on specific non‐linear functions of interest for modelling species–environment relationships, and to couple these with error distributions that are most suitable for ecological field data. Note that the identity link is our preference for these models and, in our experience, works well in practice. The *senlm* R package is an open‐source work in progress. Further contributions/improvements are welcome. We recognise that other packages (or combinations of packages) may be used to fit non‐linear statistical models (e.g., Bolker et al., 2013), although many require sophisticated coding skills. Our *senlm* package is intended to simplify and enhance ecologists' toolkit for obtaining direct parametric models of species–environment non‐linear relationships for data types commonly encountered in broad‐scale macro‐ecology.

## CAVEATS AND PITFALLS

As with many optimisation problems, the choice of optimiser and initial values can affect resulting parameter estimates. The *senlm* package takes a practical empirical approach—initial values for parameters in $\theta_{M}$ are estimated using the method of moments from splines. Initial values for other parameters depend on the model being fitted; further details can be found in the section entitled ‘Initial Parameter Estimates’ (and the ‘Init.R’ file of the *senlm* code) available on GitHub. For optimisation, the *senlm* package uses simulated annealing *via* the function mle2() with arguments optimiser = “optim” and method = “SANN” in the R package ‘*bbmle*’ (Bolker et al., 2021). A quasi‐Newton method is subsequently used to further refine these estimates, or in the event of failure (optimiser = “nlminb”).

One motivation for developing this framework was to enable estimation of useful parameters for comparative purposes. However, different mean functions (e.g., sech, HOF, beta) generally have different parameters, so to track changes in a parameter (such as *m*) through time or space or across species, it is advisable to stick with a single mean + error combination (e.g., sech + ZINBL) for the full suite of data being analysed.

Finally, a note of caution regarding rare species: the utility of any given *senlm* model will depend on there being some reasonable number of non‐zero values in the response data vector. Although the *senlm* R package will provide estimates of parameters even if there is only one non‐zero value(!), this clearly represents a case where scepticism is warranted. Simulations suggest that sample sizes of n≤20 yield parameter estimates that are highly variable, particularly for asymmetric responses (see ‘Comparison with splines’ below). It is a topic for future research to identify the percentage (or number) of non‐zero values required to construct adequate formal models of a species' response. A practical rule of thumb may lie somewhere between 1% and 5%, depending on the overall sample size. We leave this decision to the experimenter.

## EXAMPLE

### North‐east Pacific groundfish species *versus* depth

We demonstrate the utility of the method *via* analyses of North‐east Pacific groundfish species along a depth gradient. Data were obtained from the West Coast Groundfish Bottom Trawl (Slope and Shelf Combination) Survey, conducted annually by the National Oceanic and Atmospheric Association (NOAA)’s Northwest Fisheries Science Center (NOAA Fisheries, NWFSC/FRAM, 2725 Montlake Blvd. East, Seattle, WA 98112) and available online (https://www.nwfsc.noaa.gov/data/map).

We focused on count data obtained from 1999 to 2004, inclusive. We removed trawls that were not identified as ‘satisfactory’ in the database, and also removed trawls whose swept area was more extreme than the inter‐quartile range (1.682–2.025 ha) to ensure commensurability. This yielded a total of 1280 trawls, from which 239 fish taxa were identified to species. Many species were too rare to permit formal modelling (69 species occurred in only one or two trawls). We fit individual species‐specific *senlm* models to a sub‐set of 137 species that occurred in at least 7 trawls (i.e., present in ≥ 0.5% of the 1280 trawls). For each of these 137 species, we fit all combinations of six potential mean functions: {uniform, Gaussian, mixed Gaussian, beta, sech, HOF} and eight potential error distributions for count data: {Poisson, NB, ZIP, ZINB, ZIPL, ZINBL, ZIPL.mu, ZINBL.mu}. The best model for each species (out of 48 possible models) was then identified using AICc.

More than 91% of the fish species (125 of 137) required some type of non‐Gaussian shape to characterise their mean response along the depth gradient, and either a sech or mixed Gaussian model were found to be most suitable (lowest AICc) for the majority (60%) of species examined (i.e., 82 of 137; Table 2). Distributions of ΔAICc values from models fitted using different *senlm* mean functions (with the error distribution from the best AICc model) indicated that either the sech or HOF mean functions would likely be suitable for modelling most of these species (Figure S1). More than 85% of the species (117 of 137) had lower AICc values for models with NB (rather than Poisson) error distributions. In addition, the AICc best model for 73% of the species (101 of 137) included zero‐inflation, and 90% of these (91 of 101) indicated that linkage to the mean was most useful to model excess zeros (Table 2).

**TABLE 2:** Tallies of the best non‐linear model (i.e., having the lowest AICc value), constructed using a given mean function (columns) coupled with a given error distribution (rows), for the responses of each of 137 fish species (counts from 1280 trawls) *versus* a depth gradient in the Northeast Pacific

	Mean functions							
Error Distributions	Sech	Mixed Gaussian	HOF	Uniform	Gaussian	Beta	Total	Percentage
ZINBL.mu	25	9	11		5	3	**53**	38.69%
NB	2	12	2	12	2		**30**	21.90%
ZINBL	10	8	5		3	3	**29**	21.17%
Poisson	1	1		3	1		**6**	4.38%
ZIPL	2	3	1				**6**	4.38%
ZINB	1	2		1	1		**5**	3.65%
ZIP		3		2			**5**	3.65%
ZIPL.mu		3					**3**	2.19%
**Total**	**41**	**41**	**19**	**18**	**12**	**6**	**137**	
Percentage	29.93%	29.93%	13.87%	13.14%	8.76%	4.38%		

**FIGURE 1:**
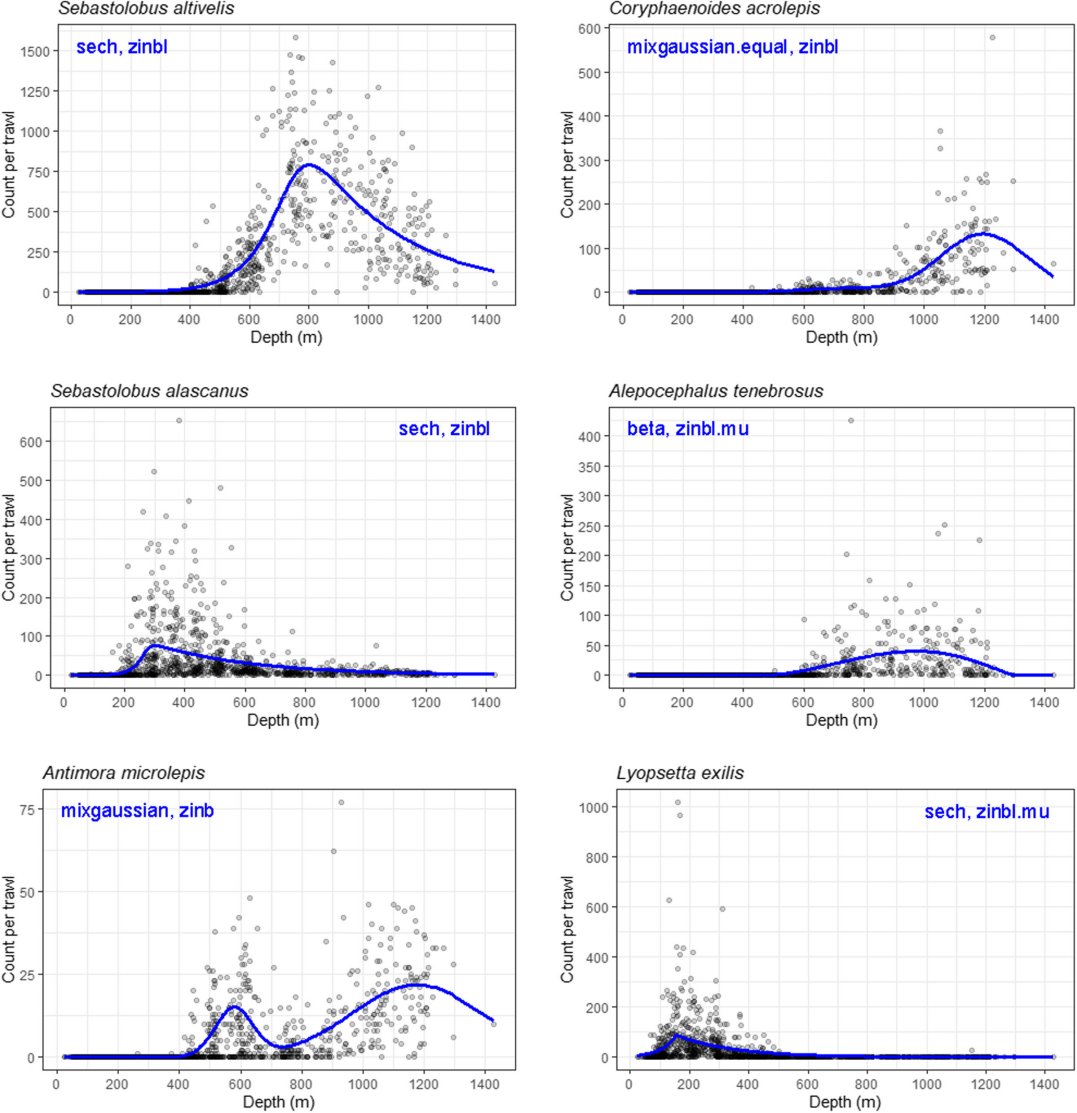
Scatter plots showing patterns of counts per trawl for each of six species of fish *versus* depth (in metres) from NOAA's North‐east Pacific dataset, with the AICc best non‐linear model, in each case, shown in blue. The modal nature of observed abundance values along the depth gradient is apparent in all cases, as is a preponderance of zero counts, particularly when mean abundances are low. Note that some of these model curves are asymmetric, bimodal, plateaued or peaked, all fairly typical of species' mean responses to a gradient and for which the suite of general mathematical functions of the *senlm* model framework have been designed to accommodate.

Patterns in abundances (counts of individuals) per trawl for individual species *versus* depth (in m) showed all of the salient features we aimed specifically to accommodate in our model framework (e.g., Figure 1). Namely, within the sampled gradient's range (i.e., the sampling frame): (i) there is a modal depth, where larger abundances of a given species occur (e.g., *Sebastolobus altivelis*); (ii) in some cases, there is more than one mode (e.g., *Antimora microlepis*); (iii) asymmetry can occur either to the right (*Sebastolobus alascanus*) or left (*Coryphaenoides acrolepis*); (iv) the modal range may be broad (platykurtic, *Alepocephalus tenebrosus*) or narrow (*Lyopsetta exilis*) and (v) there is, in almost all cases, an excess of zeros (Figure 1). These features were all handled capably by *senlm* models (Figure 1; Table S2).

**FIGURE 2:**
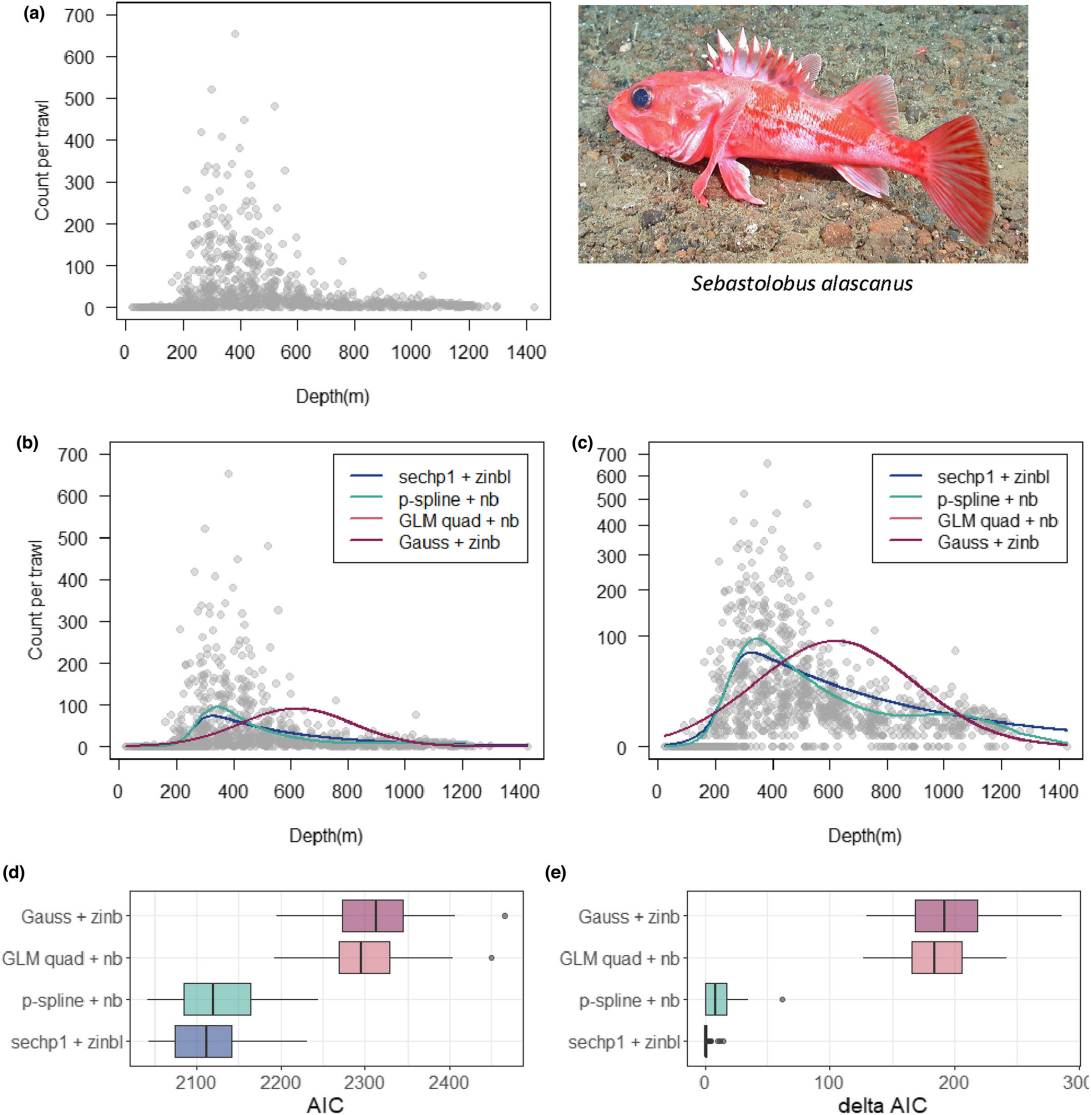
Scatter plot of counts per trawl for *Sebastolobus alascanus* (pictured) *versus* depth (in metres) shown (a) on its own and also shown with fitted lines corresponding to each of four non‐linear models (colours), with the y‐axis being either (b) on a raw abundance scale or (c) on a square‐root‐transformed scale to better see patterns for smaller values. The four models are indicated on each plot as: the *senlm* sech (*p* = 1) mean function with ZINBL errors (blue); a GAM (P‐spline) with NB errors (green); a GLM with quadratic mean function (log link) with NB errors (peach) and a Gaussian mean function with ZINB errors (burgundy), as indicated. Note that the quadratic GLM and the Gaussian model are effectively identical here (peach line is not discernible from the burgundy line). Distributions of: (d) AIC values and (e) ΔAIC values from fitting each model to 50 jackknife samples of the data. Each jackknife sample was a random proportional depth‐stratified sample of one‐quarter of the data. (*Photo image provided courtesy of Milton Love, University of California, Santa Barbara*).

We may focus on a single species (*Sebastolobus alascanus*, Shortspine thornyhead) as an exemplar for comparative purposes (Figure 2). The *senlm* model (sech [*p* = 1] mean function with ZINBL errors, AIC = 8512) provided a better model (lower AIC) than: (i) a quadratic GLM (log link) with either NB errors (AIC = 9233) or ZINB errors (AIC = 9235); (ii) a Gaussian mean function with ZINB errors (AIC = 9235) or (iii) a GAM (P‐spline) model with NB errors (AIC = 8538). Distributions of AIC (and ΔAIC) values obtained from fitting these four models to each of 50 random jack‐knife sub‐samples from the original data (where each jack‐knife sample contained one‐quarter of the data, drawn in a proportional depth‐stratified manner to cover the gradient), showed that, for this species, the *sech* and P‐spline models consistently out‐performed Gaussian or quadratic GLM models (Figure 2d, e), and that the *sech* model (marginally) out‐performed the flexible GAM model as well (Figure 2e).

The *senlm* models also outperformed these three other potential models (Gaussian, quadratic GLM and P‐spline) for all other fish species shown in Figure 1, based on AIC (Table S3). Essentially, Gaussian models or quadratic GLMs failed to identify correct modal positions along the gradient, while P‐splines typically identified modal positions rather well but did not accommodate excess zeros as well as *senlm* models.

**FIGURE 3:**
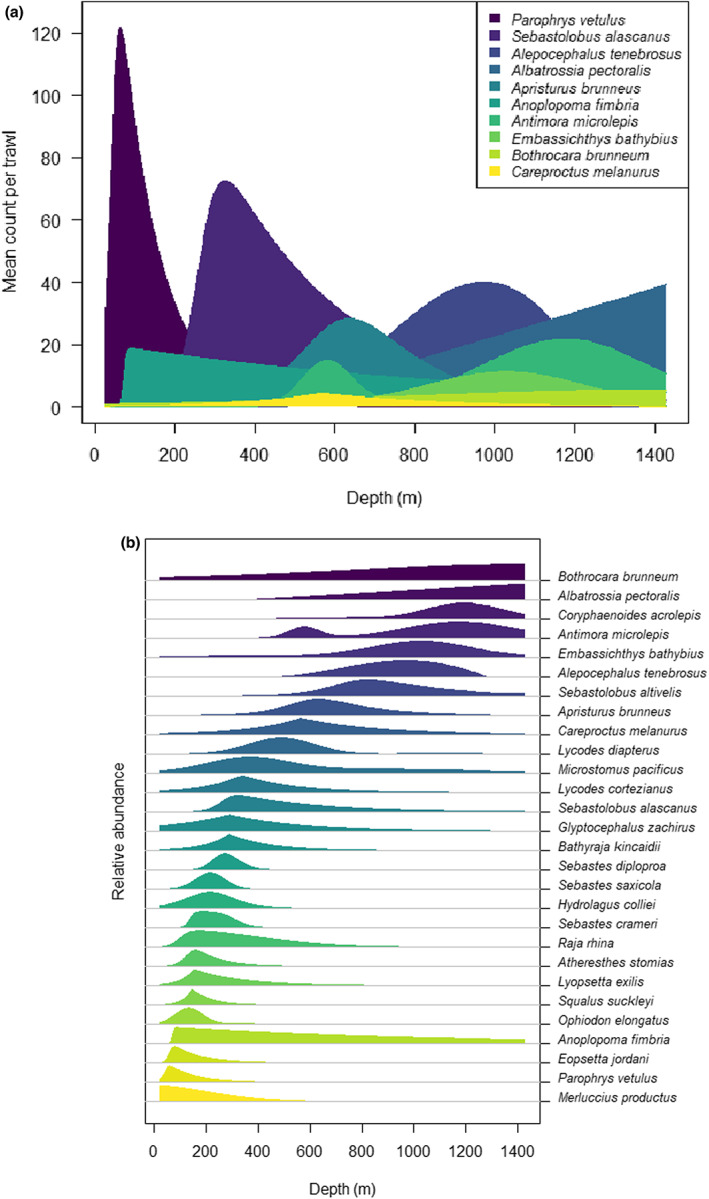
(a) Estimated mean abundance per trawl for each of 10 prominent fish species versus depth (in metres) obtained using individual AICc best *senlm* models. The layering of the species response curves (and their associated ‘viridis’ colour scale) are ordered by decreasing maximum height ($\hat{H}$). (b) Estimated relative mean abundance per trawl (as a fraction of the maximum) for the top 28 most frequently occurring fish species (in at least 20% of the samples) from the NOAA dataset extract (1999–2004) *versus* depth (in metres) obtained using individual AICc best *senlm* models. Colour and vertical ordering of the species (from top to bottom) is by decreasing modal position of their mean response along the depth gradient.

**FIGURE 4:**
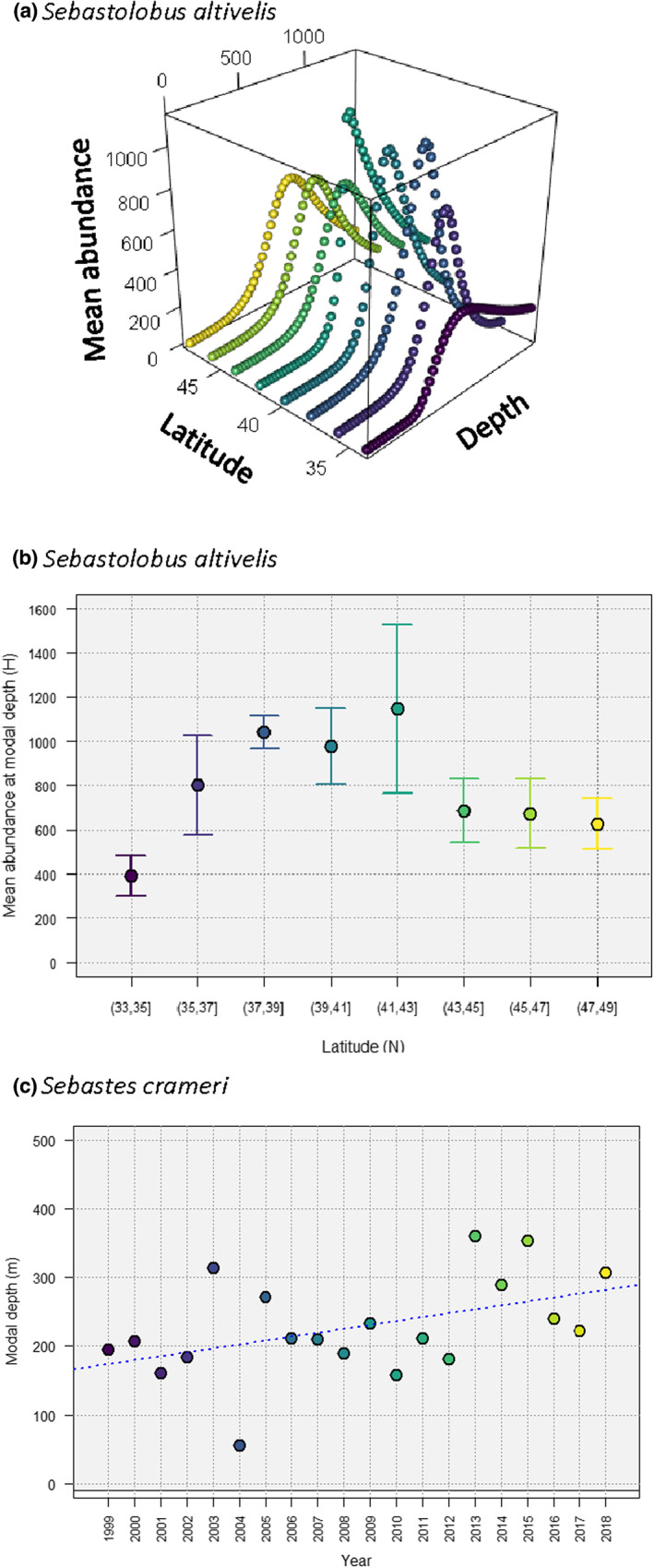
(a) Mean *senlm* response curve for *Sebastolobus altivelis versus* depth, estimated separately in each of eight 2‐degree latitudinal bins along the U. S. west coast using the sech mean function with ZINBL errors; (b) plot of the estimated peak abundance ($\hat{H}$ ± 1 jack‐knife standard error) *versus* latitude (°N) for the models shown in (a); and (c) relationship between the estimated modal position ($\hat{m}$) along the depth gradient for the mean response of *Sebastes crameri* (modelled using the sech mean function with ZINB errors) *versus* time (years). The blue dotted line shows the fitted linear regression.

Mean functions from *senlm* models fitted to a set of individual species may be drawn in multi‐species plots to characterise changes in fish communities along a gradient (Figure 3; Table S4), using either absolute (Figure 3a) or relative mean abundances (Figure 3b). In addition, *senlm* models for a single species can be fitted in a variety of different contexts to explore and quantify potential changes in species' responses to a gradient (and hence, in *senlm* parameters) through space, time, or along some other environmental parameter or factor of interest. For example, constructing separate *senlm* models of *Sebastolobus altivelis* (Longspine thornyhead) *versus* depth at different latitudes (2‐degree bins) along the western US coastline (Figure 4a) showed that the greatest estimated values of peak mean abundance per trawl (${\hat{H}}_{m}$ ~1000 individuals) occurred between latitudes ~37–43° N (Figure 4b), spanning a region of pronounced upwelling (Cape Mendocino; Jacox et al., 2018).

Species' responses to natural gradients may also vary through time, due to climate change. For example, we fit separate *senlm* models of *Sebastes crameri* (Darkblotched rockfish) obtained from the NOAA database (filtered in the same way as previously described), but for the years 1999–2018, inclusive. There has been a significant increase in the estimated position along the depth gradient at which this species reaches its peak mean abundance (*m*, the modal depth) over the past 20 years (Figure 4c, *F*_1,18_ = 6.70, *p* = 0.019). A retreat to greater (deeper) depths, as documented here, may signal a physiological response to warming surface temperatures (e.g., Kingsbury et al., 2020; Rijnsdorp et al., 2009).

## COMPARISON WITH SPLINES

One may fit non‐linear shapes to any (*X*, *Y*) data using a variety of flexible empirical methods, such as non‐parametric GAMs or P‐splines. From the resulting fitted curve one may derive estimates of certain properties of interest, such as the maximum fitted value, which may correspond to the mode. However, standardising degrees of freedom (spacing of knots, penalties, etc.) to make comparison across multiple datasets or species may not be straightforward. An extensive comparison of the statistical properties of non‐linear curve‐fitting methods is beyond the scope of this contribution. However, we performed a modest simulation study to compare the ability of spline‐based tools versus our proposed parametric *senlm* models to recover modal characteristics of species under several pertinent scenarios (see ‘Simulation_study_S4.pdf’ in Supporting Information). Simulations also permitted us to empirically estimate the coverage of confidence intervals (CIs) built using Fisher's Information matrix for *senlm* models.

First, for symmetric mean response, we found that there was greater variance in the estimated mode obtained using B‐splines or P‐splines compared to the parametric models (Gaussian or sech), even for large sample sizes (n≥100). Second, when the mean response was asymmetric, both the spline approaches and Gaussian models yielded biased estimates for modal position (m) and height (H)—specifically, m was dragged towards the larger tail and H was under‐estimated, while estimates from *sech* models were unbiased and also had lower variance. Furthermore, both the bias and the variance of m and H increased for spline approaches with increases in zero‐inflation, while estimates from *sech* models with ZI errors remained unbiased (see ‘Simulation_study_S4.pdf’ in Supporting Information for details). Finally, we found empirical coverage of CIs for either m or H, built using Fisher's Information matrix in *senlm* models, readily converged to the nominal 95% level for moderate sample sizes (Figure S4.4 in Simulation_study_S4.pdf, Supporting Information). Overall, these results suggest that spline‐based methods, although clearly more flexible than parametric models, are also more strongly affected by idiosyncrasies of individual datasets.

## DISCUSSION

Despite ample evidence for the prevalence of non‐linear (modal) species' responses to environmental (and spatial) gradients (e.g., Austin, 1976, 1980; Oksanen & Minchin, 2002; ter Braak, 1985, 1996; Whittaker, 1956, 1967), there have been surprisingly few attempts to develop a suitably flexible parametric modelling framework to characterise these for abundance data. Although mathematically elegant, the utility of symmetric modal distributions (such as Gaussian curves) for modelling species' responses tends to be the exception, rather than the rule, for most real ecological datasets (e.g., Table 2). Although non‐parametric GAMs are useful to help visualise the general non‐linear pattern of responses along a gradient (e.g., Yee, 2015; Yee & Mitchell, 1991), they do not yield ecologically interpretable parameters. Furthermore, estimates of modal parameters derived from spline‐based models were found to be biased in simulations where the mean species' response was asymmetric (Supporting Information S4).

We have provided here a suite of non‐linear mathematical functions for the mean response of species to environmental gradients that yield interpretable parameters, yet flexibly accommodate asymmetry, peakedness/flatness or bi‐modal shapes—features that are empirically readily apparent in natural systems. A non‐linear function for the mean response must be coupled with an appropriate error distribution. Importantly, the mean function and the error distribution work together in *senlm* models to capably track the overall shape of the species' response appropriately. Most species display excess zeros (e.g., Table 2). Furthermore, in our example, almost all models requiring zero‐inflated errors worked best when the probability of an excess zero was linked to the mean response. This clearly supports the genuine utility of the linked models offered in the *senlm* framework (e.g., ZIPL, ZINBL, ZINBL.mu, ZIGL, etc.).

The suite of error distributions offered by the *senlm* package caters to a large array of data types commonly used in ecology under a single umbrella. We have also tailored certain known statistical distributions (e.g., beta) to accommodate ecological applications better (e.g., ZITAB). In practical terms, using the *senlm* R package, the end‐user may simply identify the data type (e.g., discrete, continuous, cover, etc., see Table 1), then information criteria can be used to choose among competing potential models for that data type.

We found for the example dataset that three of the available mean functions (sech, mixed Gaussian and HOF) modelled the majority of species rather well. Some species were better modelled, however, using a beta mean function, usually when the mean response was fairly flat, or when there were hard upper/lower bounds on a species' occurrence along the gradient. In contrast, the sech function did well when the mean response was more peaked. Development of a single mean function that can be adjusted parsimoniously to model the majority of mean response shapes is clearly desirable.

We consider the *senlm* model framework presented here to be merely the beginning—it provides a core parametric approach for modelling one species along one environmental gradient—upon which more complex models may be built. Future developments might provide for: (i) inclusion of additional factors, random effects or interactions; (ii) non‐linear models of species' responses along two or more gradients (*X* variables) simultaneously (a species' realised environmental niche); (iii) multivariate non‐linear models of two or more species (*Y* variables) simultaneously; (iv) methods to integrate errors in parameters (including autocorrelation) through space and time, perhaps using an hierarchical Bayesian approach. Associations among species might also be modelled formally (e.g., Anderson et al., 2019; Ovaskainen & Abrego, 2020; Warton et al., 2015); positive (or negative) relationships could arise from species having similar (or dissimilar) fitted responses to the gradient, while other types of inter‐species associations may be evident across their residuals.

We invite contributions to the *senlm* package to enhance its utility across a broader range of applications. For example, with increasing interest in climate change, non‐linear curves explicitly identifying species' tolerances to temperature (including ‘tipping points’, e.g., see Kenek et al., 2011) would be welcome. Other error distributions could also be added, such as generalised Poisson distributions (Aitchison & Ho, 1989; Clarke et al., 2006; Coly et al., 2016), truncated distributions (e.g., Nadarajah & Kotz, 2006), distributions catering to under‐dispersion (Rogers, 1974) or hurdle models (Mullahy, 1986; Zeileis et al., 2008). Extensions to model functional traits along gradients are also desirable.

We anticipate that the *senlm* framework will empower ecologists to construct bespoke non‐linear models for species of every conceivable type (e.g., plankton, invertebrates, microbes, trees, fish, birds, mammals, etc.). Our aim is for large datasets covering a variety of places, times and taxonomic groups to be tackled with confidence, regardless of how abundance has been quantified (e.g., biomass, counts, cover, presence/absence, etc.), allowing rigorous quantification of individual species–environment relationships.

## AUTHOR CONTRIBUTIONS

MJA developed all of the conceptual ideas for the model framework, led the research program, wrote and ran code for all simulations and wrote the manuscript and supplements; DCIW developed the new parameterisation of the beta distribution and wrote R code for the *senlm* package; WLS developed the new parameterisation of the sech function; AP wrangled data, developed the R package with DCIW, developed code and ran analyses to create Table 2 and helped create some of the Figures. All authors contributed refinements to the final draft of the manuscript.

### PEER REVIEW

The peer review history for this article is available at https://publons.com/publon/10.1111/ele.14121.

## Supporting information


Vignette S1.pdf

Data S1

Rcode S1.txt

Figure S1.pdf

Table S2

Table S3

Table S4

Simulation study S4.pdf

## Data Availability

Data used in examples are available on Dryad at DOI https://doi.org/10.5061/dryad.c59zw3rbp. The R package is provided on GitHub at: https://primer‐e.github.io/senlm/.
