# Supplementary material for: Non‐linear models of species' responses to environmental and spatial gradients
Source: Ecol Lett. 2022 Oct 21;25(12):2739–52. doi: 10.1111/ele.14121 (PMC9828393; doi:10.1111/ele.14121)
Supplement: Supplementary file 1 — Vignette S1.pdf [file ELE-25-2739-s004.pdf]

Vignette_S1.pdf, Supporting Information

A vignette to accompany the R package ‘*senlm*’

1. Introduction

An R package called *senlm* (“species-environment non-linear models”) is available at the following GitHub repository: https://primer-e.github.io/senlm/). The purpose of this package is to implement a framework to model the nonlinear response of a single species (*Y*) to a single environmental gradient (*X*) using a parametric maximum likelihood (ML) approach. Any individual model is comprised of two elements: a *mean function* and an *error distribution*. A list of the mean functions and error distributions currently available, along with mathematical details, are found on the Github website. Our purpose here is to provide a vignette to demonstrate the use of the package. Two additional files are found as Supporting Information and are used here:

`Data_S1.csv`: Excerpt[1] of NOAA groundfish annual trawl data containing trawl ID, area swept, latitude, longitude, depth, project name and counts of abundances for each of 310 fish species for the years 1999-2018; and

`Rcode_S1.txt`: R code to accompany this vignette.

2. Fitting a model using *senlm*

We shall begin by installing the package. Note that the following 2 lines do not need to be run again once the package has been installed.

```
install.packages("devtools")
devtools::install_github("PRIMER-e/senlm")
```

Load the senlm R package.

```
library(senlm)
```

Next, we shall load a dataset into R. The name of the file is “Data_S1.csv”, and it is found as Supporting Information.

```
fish <- read.csv(file.choose(), row.names = 1 )
head(fish)
```

Our interest here is to model the response of a species (counts of abundances) along the depth gradient. Although this is a spatial gradient, it acts as a proxy for a host of important environmental variables (pressure, temperature, light, etc.) that change dramatically with

[1] This excerpt was created by filtering the raw NOAA data (available from https://www.nwfsc.noaa.gov/data/map) as follows: (a) we removed trawls not marked as ‘satisfactory’; (b) we removed trawls whose swept area was either missing (recorded as ‘NA’), or was outside the interquartile range (1.57 – 2.01 ha) to ensure commensurability; (c) we removed trawls not belonging to one of the following projects: ‘Groundfish Slope Survey’, ‘Groundfish Shelf Survey’ or ‘Groundfish Shelf and Slope Combination Survey’; (d) we retained only the years 1999 – 2018; (e) we retained only fish species that occurred in at least one trawl and that were identified to species level.

increasing depth in the ocean. For simplicity in what follows, we shall rename the depth variable in the data frame.

```
colnames(fish)[5] <- "depth"
```

For starters, let's focus on a single species: *Sebastolobus alascanus*. We shall also remove any rows with missing values (‘NA’), and will further simplify the column names to "x" and "y".

```
dat1 <- na.omit(subset(fish, select = c(depth, Sebastolobus.alascanus) ) )
colnames(dat1) <- c("x", "y")
```

It is always a good idea to consider a scatter plot in the first instance to get a feel for the data. Here is a simple scatter plot, with transparency in the fill of the points to make it easier to see overlaps.

```
plot(dat1$x, dat1$y, las = 1, xlab = "Depth (m)",
     ylab = "Count per trawl", ylim = c(0,800))
mygrey <- grey(level = 0.65, alpha = 0.4)
points(dat1$x, dat1$y, pch = 21, col = mygrey, bg = mygrey)
```

For this species, we can see here a clear unimodal response that is asymmetric. There also appears to be a large number of zero values all along the depth gradient.

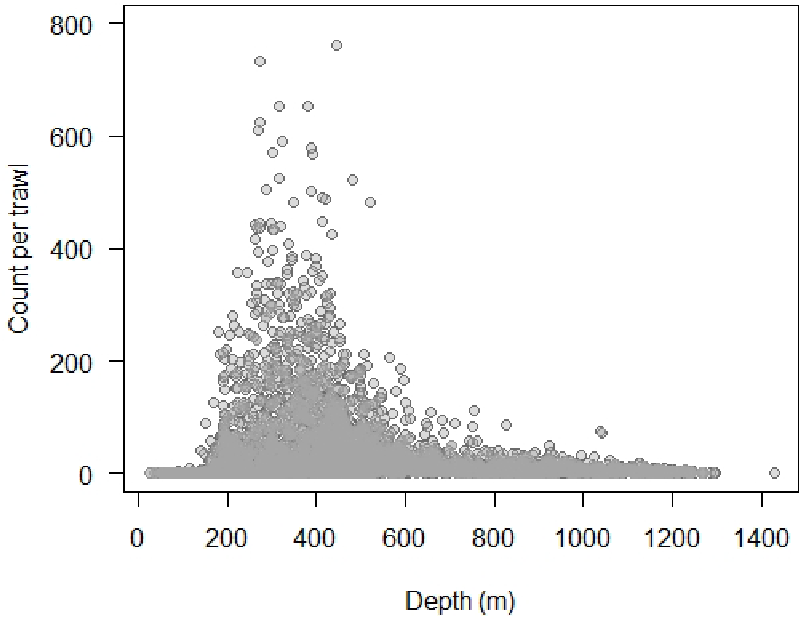

Let's fit one of the *senlm* models. Specifically, we shall fit a sech mean function and a zero-inflated negative binomial (ZINB) error distribution. A short-hand way of writing this model is “sech + zinb”.

```
sech.zinb <- senlm(data = dat1, xvar = "x", yvar = "y",
                   mean_fun = "sech", err_dist = "zinb")
```

Note that running the above model might take a minute, simply due to the sheer size of the data file. There are over 5000 observation rows (trawls). Once the model has run, we can summarise

the model object. This summary shows the estimated model parameters and information criteria for this model.

```
summary(sech.zinb)
```

```
$model
[1] "sech_zinb"

$theta
          H           m           s           r           p          pi 
 98.25346555 286.89330543  56.22991413   0.92464306   3.03732330   0.07785348 
        phi 
  1.86828121 

$IC
    npar      nll      AIC     AICc      BIC 
    7.00 16193.81 32401.62 32401.64 32448.52 
```

The model parameters, held in `sech.zinb$theta`, include both sets of parameters: those required for the mean function (these are $\theta_M = \{H, m, s, r, p\}$ for the sech mean function) and also those required for the error distribution. For the zero-inflated negative binomial, these are $\theta_E = \{\phi, \pi\}$, with ϕ (‘`phi`’ in the output) being the dispersion parameter and π (‘`pi`’ in the output) being the probability of an excess zero. We can also get 95% confidence intervals on the parameters (estimated using Fisher’s information matrix[2]). One can change the level of the confidence intervals by using `conf.level` as an additional argument in the model call. The default is `conf.level = 0.95`.

```
rbind(lower=sech.zinb$lb, upper=sech.zinb$ub)
```

```
              H        m        s         r        p         pi      phi
lower  90.50123 275.1920 45.56451 0.9112202 2.122891 0.05681997 1.717206
upper 106.66974 298.5946 69.39180 0.9361044 4.345646 0.10579975 2.032647
```

3. Plotting results

A basic plot can be drawn right away from the model object to get a visualisation of the fitted mean function along with the data.

```
plot(sech.zinb)
```

[2] Note: occasionally, due to numerical instability of the estimated Hessian matrix, the standard errors are not calculated.

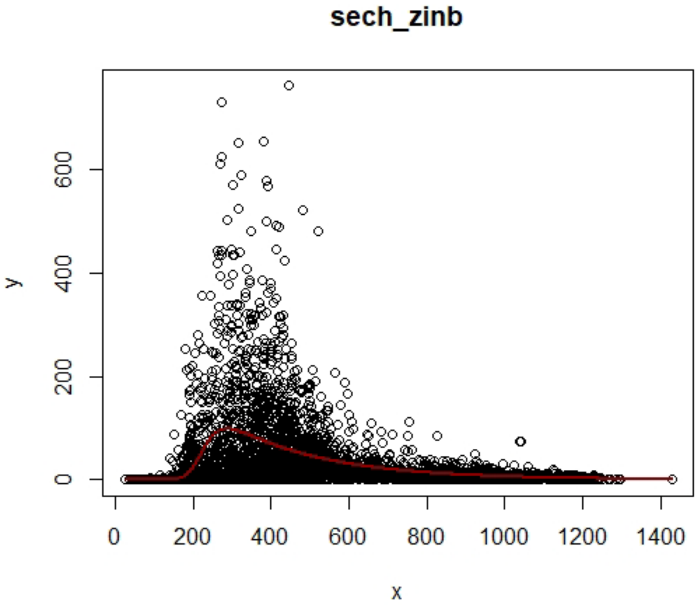

We can make this look quite a lot better with a few tweaks, including by changing the scale to square roots, in order better to see the detail for smaller abundance values, *viz*:

```
plot(dat1$x, sqrt(dat1$y), las = 1, yaxt = "n", xlab = "Depth (m)",
     ylab = "Count per trawl", ylim = c(0,sqrt(800)))
points(dat1$x, sqrt(dat1$y), pch = 21, col = mygrey, bg = mygrey)
axis(side = 2, at = sqrt(seq(0,800, by = 100)),
     labels = seq(0,800, by = 100), las = 1 )
```

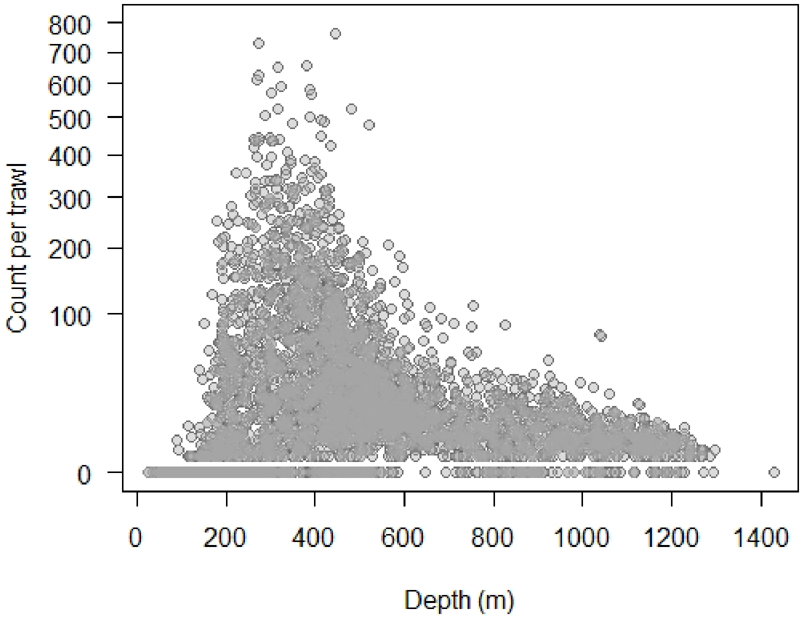

Let's add the fitted line, and also the values for two parameters of interest: *H*, the peak in mean abundance, and *m*, the modal position of that peak along the gradient.

```
# add the fitted line:
```

```
predict.x <- seq(from=min(dat1$x), to = max(dat1$x), length.out = 100000)
yfit.sech <- predict(sech.zinb, predict.x)
lines(predict.x, sqrt(yfit.sech), col = "#224188", lwd = 2)

# add on the estimated values for m and H for this model
m = sech.zinb$theta["m"] ; H = sech.zinb$theta["H"]
axis(side = 1, at = m, label = "m", col.axis = "red", col.ticks = "red")
lines(x = c(m, m), y = c(-100,sqrt(H)), col = "red",
      lwd = 2, lty = "dashed")
lines(x = c( -100, m), y = c(sqrt(H), sqrt(H)), col = "red",
      lwd = 2, lty = "dashed")
text (x = 0, y = sqrt(120), "H", col = "red")
```

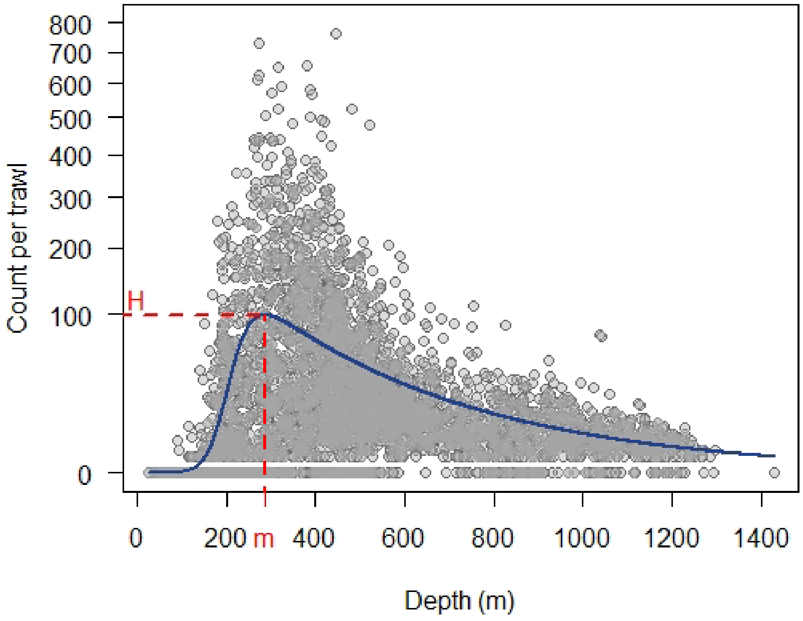

As indicated in the summary of the model output and shown on the plot above, the modal position of *Sebastolobus alascanus* along the depth gradient is estimated by the sech + zinb model to be at m = 287 metres (with 95% CI of 275 – 299 metres), and its peak mean abundance at that modal position (excluding excess zeros) is estimated to be ~ H = 98 individuals (with 95% CI of 90 – 107 individuals).

Note also that the blue line in the above figure is tracing the non-linear mean (μ_i), which is the mean of the NB distribution within the ZINB mixture. For error distributions that do not have excess zeros (implicitly, $\pi = 0$), the expected value of the response variable is equivalent to this (i.e., $E(Y_i) = \mu_i$). However, for zero-inflated models, such as this one, $E(Y_i) = (1 - \pi)\mu_i$. We can also obtain values for the expectation of the response variable that *includes* excess zeros, thus:

```
theta <- sech.zinb$theta
E.y <- (1 - theta["pi"]) * yfit.sech
lines(predict.x, sqrt(E.y), col = "#FFA500", lwd = 2)
```

```
# A legend:
mylines = c("excluding excess zeros", "including excess zeros")
legend(x = 600, y = sqrt(800), title = "Mean abundance", cex = 0.8,
       legend = mylines, lwd = 2, col = c("#224188","#FFA500"))
```

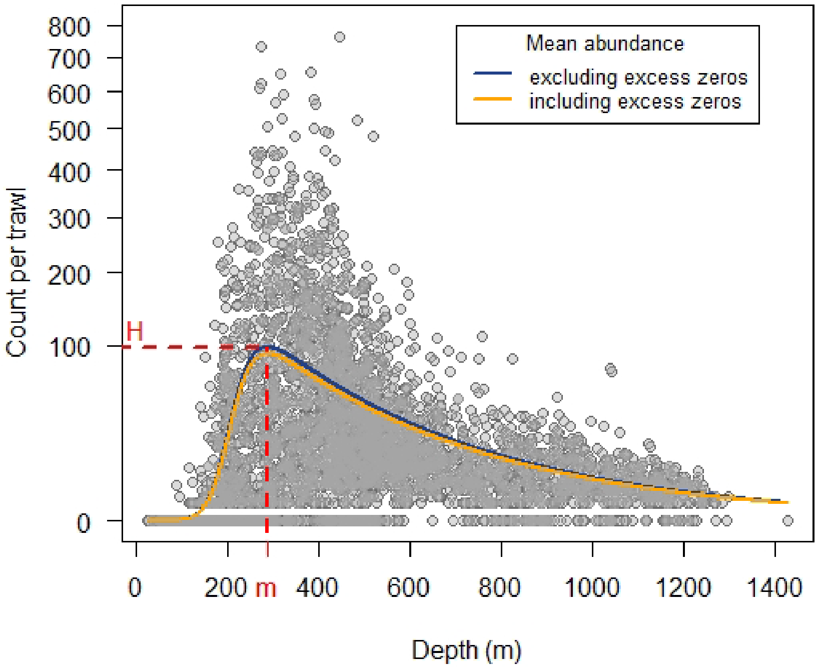

In this case, there is only about a 7% reduction in the mean, and the effect of excess zeros on expected values also applies uniformly along the gradient for this type of model (i.e., we have a single value for π when we use a ZINB error distribution). Thus, the expected mean abundance *per trawl* at the species' modal position $x_i = m$ that *includes* excess zeros is estimated as $E(Y|x_i = m) = (1-\pi)H$. Plugging in our estimates for H and π, we have

```
# expected mean abundance per trawl at m
H.y <- (1 - theta["pi"]) * theta["H"]

# 95% CI for this
H.y.low <- (1 - theta["pi"]) * sech.zinb$lb["H"]
H.y.up <- (1 - theta["pi"]) * sech.zinb$ub["H"]

# result
EY.at.m <- c(H.y, H.y.low, H.y.up)
names(EY.at.m) <- c("expected.val","95% lower","95% upper")
EY.at.m
```

```
expected.val    95% lower    95% upper
    90.60369     83.45488     98.36487
```

So, our estimated expectation for the number of individuals per trawl at the modal depth (*including* excess zeros) is ~ 91 individuals (with 95% CI of 83 – 98 individuals).

4. Comparing model fits visually

It is natural to wish to compare the *senlm* models with other potential models that have classically been used to model nonlinear responses of species to gradients. For example, let's consider what a quadratic GLM model with ZINB errors would look like for these data.

```
library(pscl) # has zeroinfl() function
glm.zinb <- zeroinfl(data = dat1, y ~ x + I(x^2) | 1, dist = "negbin")
summary(glm.zinb)
```

```
Call:
zeroinfl(formula = y ~ x + I(x^2) | 1, data = dat1, dist = "negbin")

Pearson residuals:
     Min       1Q   Median       3Q      Max 
-0.52412 -0.52405 -0.49559  0.07203 12.69954 

Count model coefficients (negbin with log link):
              Estimate Std. Error z value Pr(>|z|)
(Intercept)  4.065e+00        NaN     NaN      NaN
x            1.446e-03        NaN     NaN      NaN
I(x^2)      -3.504e-06        NaN     NaN      NaN
Log(theta)  -4.130e-01        NaN     NaN      NaN

Zero-inflation model coefficients (binomial with logit link):
            Estimate Std. Error z value Pr(>|z|)
(Intercept)   -0.178        NaN     NaN      NaN

Theta = 0.6617 
Number of iterations in BFGS optimization: 11 
Log-likelihood: -1.835e+04 on 5 Df
```

We cannot easily compare our results with these results, as most of the model parameters differ. We may usefully compare the AIC for this “quadratic GLM + zinb” model with the AIC we obtained for the sech + zinb model above to consider the relative goodness-of-fit, given the number of parameters.

```
AIC(glm.zinb)
```

```
[1] 36714.1
```

For this dataset, the sech + zinb model clearly provides a better fit based on this criterion (AIC = 32404). What about a Gaussian curve with NB errors?

```
gauss.nb <- senlm(data = dat1, xvar = "x", yvar = "y",
                  mean_fun = "gaussian", err_dist = "negbin")
summary(gauss.nb)
```

```
$model
[1] "gaussian_negbin"

$theta
         H          m          s        phi 
235.643661 640.143960 162.182420   4.522316 
```

```
$IC
    npar      nll      AIC     AICc      BIC
    4.00 17850.94 35709.89 35709.90 35736.68
```

This “Gaussian + nb” approach (AIC = 35710) may be a slight improvement on the quadratic GLM + zinb model, but its AIC is also quite a bit larger than the sech + zinb model AIC.

While information criteria are important because they provide us with an objective way to compare models like these, a picture is worth a thousand words, and a very useful diagnostic tool is simply to compare the model fits visually for multiple candidate models in a single plot. Let's re-draw the plot and put fitted curves on it for all three models.

```
# Start with the previous code for the (sqrt-scaled) plot
# (but without the red lines showing H and m).
   plot(dat1$x, sqrt(dat1$y), las = 1, yaxt = "n", xlab = "Depth (m)",
        ylab = "Count per trawl", ylim = c(0,sqrt(800)))
   points(dat1$x, sqrt(dat1$y), pch = 21, col = mygrey, bg = mygrey)
   axis(side = 2, at = sqrt(seq(0, 800, by=100)),
        labels = seq(0, 800, by=100), las = 1 )

   # Now for the fitted lines:
   mycols = c("#224188","#CC6677","#882255")

   # Add the fitted line first for the sech model:
   predict.x <- seq(from=min(dat1$x), to = max(dat1$x), length.out = 100000)
   yfit.sech <- predict(sech.zinb, predict.x)
   lines(predict.x, sqrt(yfit.sech), col = mycols[1], lwd = 2)

   # Add to this the quadratic GLM + ZINB errors.
   yfit.glm <- predict(glm.zinb, type = "response")
   glmx <- dat1$x[order(dat1$x)]; glmy <- yfit.glm[order(dat1$x)]
   lines(glmx, sqrt(glmy), col = mycols[2], lwd = 2)

   # And then the Gaussian model with NB errors
   yfit.gauss <- predict(gauss.nb, predict.x)
   lines(predict.x, sqrt(yfit.gauss), col = mycols[3], lwd = 2)

   # A legend:
   mods = c("sech + zinb", "GLM, quadratic + zinb", "Gauss + nb")
   legend(x = 800, y = sqrt(800), legend = mods, lwd = 2,
          col = mycols, cex = 0.8)
```

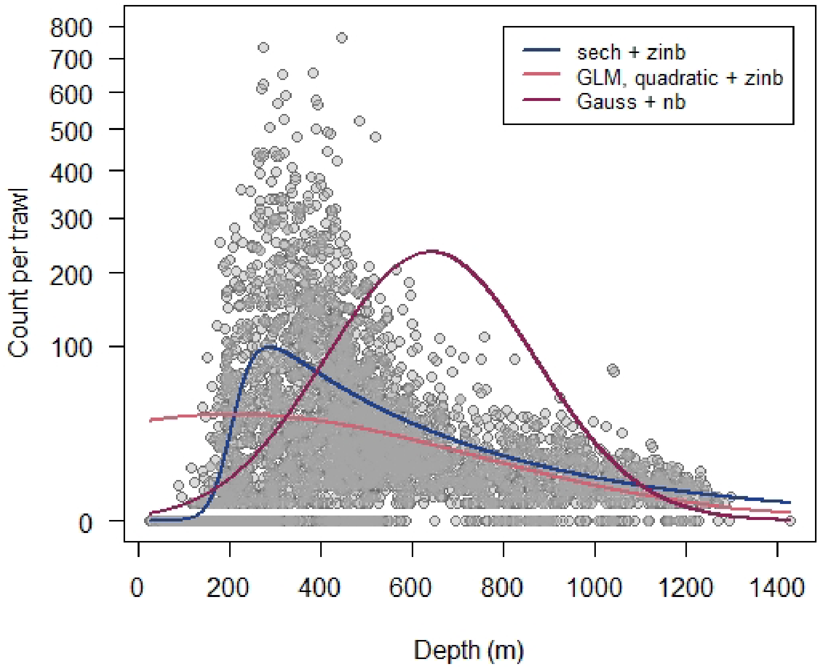

Qualitatively, there are dramatic differences between these three different approaches to modelling these data, which can really be appreciated when we see this plot. Although both the quadratic GLM and Gaussian curves are unimodal, they obviously fail to accommodate the asymmetry in the species' modal response to the gradient. More specifically, the Gaussian + nb model will clearly (in this case) over-estimate both m and H, while the quadratic GLM + zinb model will clearly under-estimate both of these parameters.

5. Comparing *senlm* models for a given data type

The above exercise obviously begs the question: which mean function and error distribution combination(s) would be most appropriate to use for a given set of data? The `msenlm()` function in the *senlm* R package provides some help in this regard. It allows multiple specified *senlm* models to be fitted to a single dataset, then provides a useful summary output for comparing them in terms of parameter estimates and information criteria to assess relative goodness-of-fit.

For this example, we will consider a different species, *Antimora microlepis*. To help speed calculations, we will also take a subset of data; specifically, for the years from 1999-2004. Here is our new data frame ("`dat2`"):

```
dat2 <- na.omit(subset(fish, year <=2004,
                select = c(depth, Antimora.microlepis) ) )
colnames(dat2) <- c("x", "y")
```

First, examine a simple scatter plot (always a good idea!)

```
plot(dat2$x, dat2$y, xlab = "Depth(m)", ylab = "Count per trawl",
     las=1, ylim = c(0,80) )
points(dat2$x, dat2$y, pch = 21, col = mygrey, bg = mygrey)
```

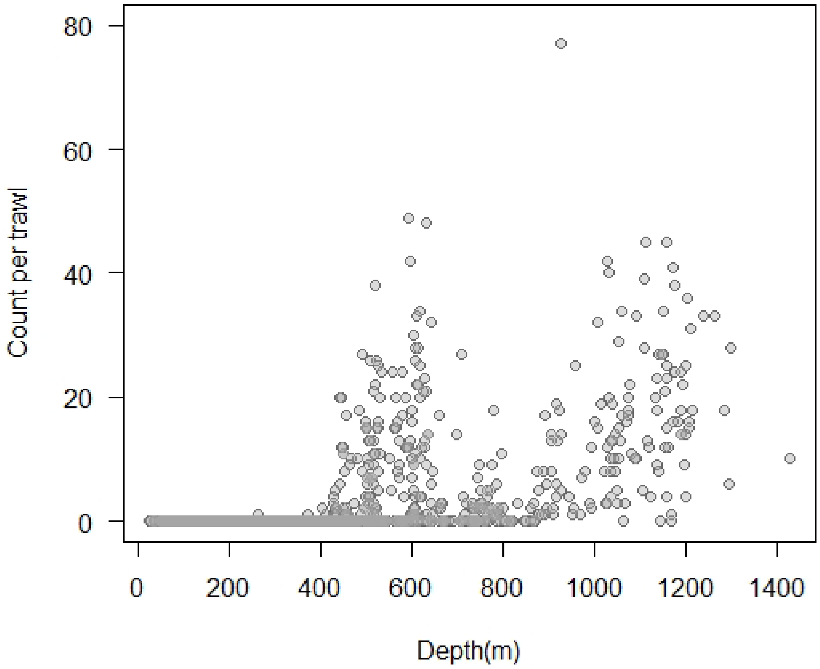

We can detect a bit of bi-modality here. The "trough" in the middle of the gradient (at a depth of around 800 m) could correspond to an "oxygen minimum zone". Let's create a list of some reasonable possible models for count data that might be used to model these data. (*Note*: of course, there are more!)

```
count.models <- set_models(mean_fun = c("sech","hofV","mixgaussian"),
                err_dist = c("poisson","zip","zipl",
                             "negbin","zinb","zinbl") )
count.models[,1:2]
```

```
      mean_fun err_dist
1         hofV  poisson
2         hofV      zip
3         hofV     zipl
4         hofV   negbin
5         hofV     zinb
6         hofV    zinbl
7  mixgaussian  poisson
8  mixgaussian      zip
9  mixgaussian     zipl
10 mixgaussian   negbin
11 mixgaussian     zinb
12 mixgaussian    zinbl
13        sech  poisson
14        sech      zip
15        sech     zipl
16        sech   negbin
17        sech     zinb
18        sech    zinbl
```

We now have an object that specifies 18 different models to pass to the `msenlm()` function. We would like to see which ones do best by reference to information criteria. (*Note*: the following line will probably take quite a few minutes to run...)

```
fits <- msenlm(models = count.models, data = dat2, xvar = "x", yvar = "y")
multi <- summary(fits)
```

The entire tabular summary will be printed in the R console when you type the above line (we shall not re-produce that here). Each line in the table corresponds to a given model fit, as indicated. Note that 'NA's appear for parameters that are not estimated in any given model.

Below is an excerpt of the full summary. It is also useful to put these summarised results in order of increasing values of (say) AICc, so that the "best" models (based on that criterion) appear at the top of the list.

```
multi[order(multi$AICc),c(4,5,6,24:28)]
```

```
                  model    mean_fun err_dist npar      nll      AIC     AICc      BIC
11    mixgaussian_zinb mixgaussian     zinb    8 1372.849 2761.698 2761.820 2802.351
6           hofV_zinbl        hofV    zinbl    8 1376.475 2768.950 2769.072 2809.604
18          sech_zinbl        sech    zinbl    8 1376.943 2769.887 2770.009 2810.540
10  mixgaussian_negbin mixgaussian   negbin    7 1380.449 2774.898 2774.993 2810.470
12   mixgaussian_zinbl mixgaussian    zinbl    9 1389.584 2797.169 2797.321 2842.904
4          hofV_negbin        hofV   negbin    6 1410.208 2832.417 2832.488 2862.907
5            hofV_zinb        hofV     zinb    7 1410.208 2834.417 2834.512 2869.989
16         sech_negbin        sech   negbin    6 1419.921 2851.841 2851.912 2882.331
17           sech_zinb        sech     zinb    7 1419.673 2853.345 2853.440 2888.917
9     mixgaussian_zipl mixgaussian     zipl    8 2259.888 4535.775 4535.897 4576.429
14            sech_zip        sech      zip    6 2603.573 5219.147 5219.218 5249.637
2             hofV_zip        hofV      zip    6 2613.087 5238.174 5238.245 5268.664
3            hofV_zipl        hofV     zipl    7 2642.641 5299.282 5299.377 5334.854
8      mixgaussian_zip mixgaussian      zip    7 2665.841 5345.682 5345.777 5381.254
15           sech_zipl        sech     zipl    7 2723.104 5460.208 5460.303 5495.780
13        sech_poisson        sech  poisson    5 3379.935 6769.869 6769.920 6795.278
1         hofV_poisson        hofV  poisson    5 3387.538 6785.076 6785.127 6810.485
7  mixgaussian_poisson mixgaussian  poisson    6 3791.501 7595.002 7595.073 7625.492
```

Apparently, the mixed Gaussian mean function, with ZINB errors, is the preferred model here (out of this suite of 18 possible models).

When we consider the results for the 18 different models, it is apparent that, for any given error distribution, the mixed Gaussian mean function generally does better than other choices for the mean function. This is in tune with (and in some sense validates) our earlier observation from the plot that there may well be a bimodal pattern of response for this species along the depth gradient.

Let's look at a summary for the "best" model (based on AICc).

```
mixg.zinb <- senlm(data = dat2, xvar = "x", yvar = "y",
                   mean_fun = "mixgaussian", err_dist = "zinb")
summary(mixg.zinb)
```

```
$model
[1] "mixgaussian_zinb"
```

```
$theta
          H           a          m1          m2          s1          s2
 20.6478075   0.4542262 571.4145595 1204.0374586  59.0368756 219.1605439
         pi         phi
  0.2244444   1.0906642

$IC
    npar      nll      AIC     AICc      BIC
   8.000 1372.849 2761.698 2761.820 2802.351
```

We can add the fitted mean function (excluding excess zeros) to the plot.

```
predict.x <- seq(from=min(dat2$x), to = max(dat2$x), length.out = 100000)
yfit.mixg.zinb <- predict(mixg.zinb, predict.x)
lines(predict.x, yfit.mixg.zinb, col = "#00BFC4", lwd = 2)
legend(x = 20, y = 80, legend = "mix.gauss + zinb",
       lwd = 2, col = "#00BFC4", cex = 0.8)
```

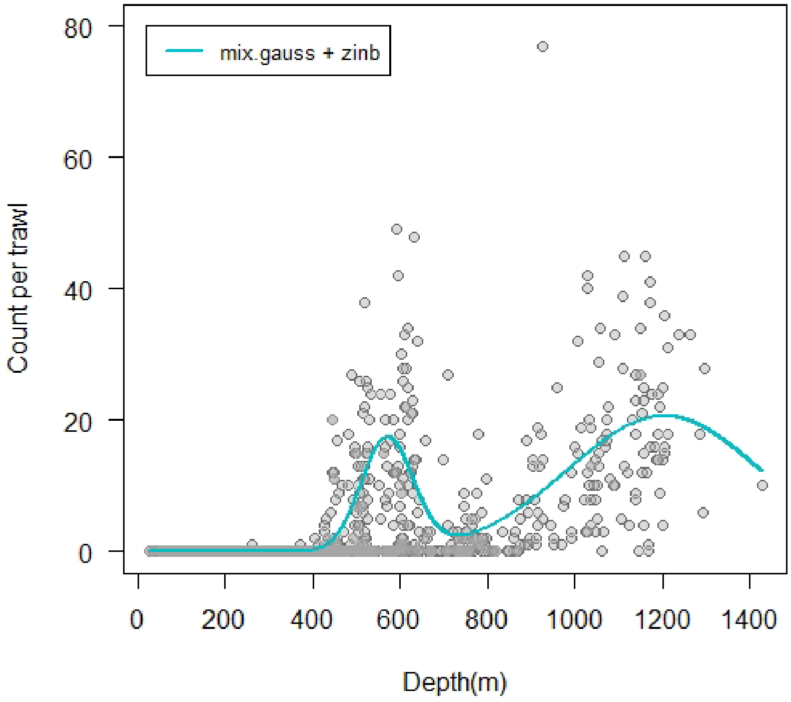

We have already seen how useful it can be to plot multiple models in a single diagram to compare them qualitatively. We may not, however, wish to look at all of the above 18 models simultaneously. However, an interesting and important point to note is that if we use different error distributions, then we can get very different fitted model curves, even if we use the same mean function. For example, let's look visually at what we would get using the mixed Gaussian mean function with a suite of different error distributions for these data.

For clarity, here are our five additional models of interest:

```
mixg.zinbl <- senlm(data = dat2, xvar = "x", yvar = "y",
                    mean_fun = "mixgaussian", err_dist = "zinbl")
```

```
mixg.negbin <- senlm(data = dat2, xvar = "x", yvar = "y",
                     mean_fun = "mixgaussian", err_dist = "negbin")
mixg.zipl <- senlm(data = dat2, xvar = "x", yvar = "y",
                   mean_fun = "mixgaussian", err_dist = "zipl")
mixg.zip <- senlm(data = dat2, xvar = "x", yvar = "y",
                  mean_fun = "mixgaussian", err_dist = "zip")
mixg.pois <- senlm(data = dat2, xvar = "x", yvar = "y",
                   mean_fun = "mixgaussian", err_dist = "poisson")
```

The fitted values for these models along the gradient are extracted from them easily.

```
yfit.mixg.zinbl <- predict(mixg.zinbl, predict.x)
yfit.mixg.zinb <- predict(mixg.zinb, predict.x)
yfit.mixg.negbin <- predict(mixg.negbin, predict.x)
yfit.mixg.zipl <- predict(mixg.zipl, predict.x)
yfit.mixg.zip <- predict(mixg.zip, predict.x)
yfit.mixg.pois <- predict(mixg.pois, predict.x)
```

Redrawing the plot with lines for all six of these models can be done as follows:

```
# Start again with the previous code for the scatter plot
   plot(dat2$x, dat2$y, xlab = "Depth(m)", ylab = "Count per trawl",
        las=1, ylim = c(0,80) )
   points(dat2$x, dat2$y, pch = 21, col = mygrey, bg = mygrey)

# Add the fitted lines with colours of your choice…
   mycols2 <- c("#F8766D","#00BFC4","#619CFF","#C77CFF","#00BA38","#CD9600")
   lines(predict.x, yfit.mixg.zinbl, col = mycols2[1], lwd = 2)
   lines(predict.x, yfit.mixg.zinb, col = mycols2[2], lwd = 2)
   lines(predict.x, yfit.mixg.negbin, col = mycols2[3], lwd = 2)
   lines(predict.x, yfit.mixg.zipl, col = mycols2[4], lwd = 2)
   lines(predict.x, yfit.mixg.zip, col = mycols2[5], lwd = 2)
   lines(predict.x, yfit.mixg.pois, col = mycols2[6], lwd = 2)

# and the legend.
   mods = c("zinbl","zinb", "negbin", "zipl", "zip", "pois")
   legend(x = 20, y = 80, legend = mods, lwd = 2, col = mycols2)
```

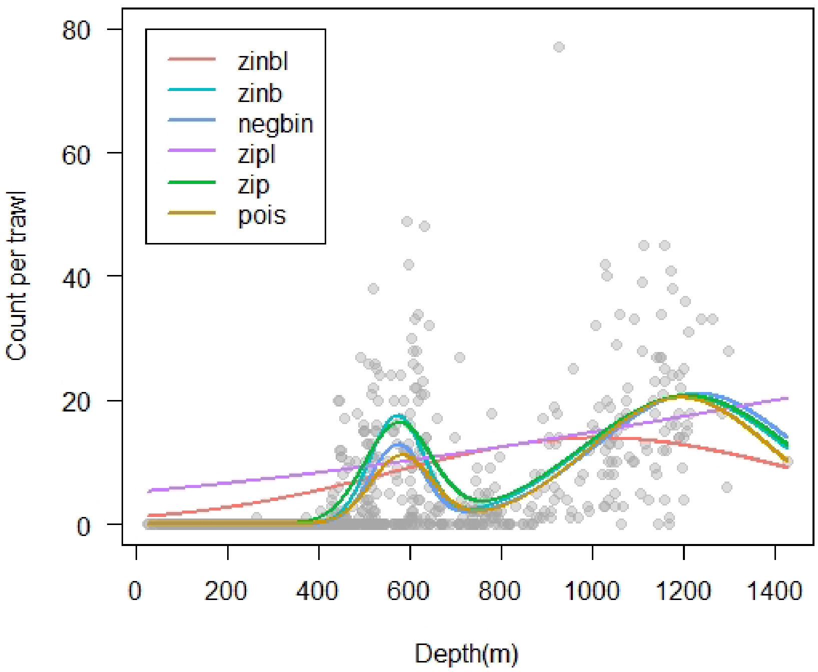

The most interesting feature here is that, for these data, the use of either of the linked error distributions (ZINBL or ZIPL) resulted in failure to detect a bimodal pattern. More generally, the take-home message here is that a poor choice for the error structure can result in counter-intuitive fitted mean functions that may potentially miss important qualitative features inherent in the response data[3].

6. Visualising error structures on plots

The above example perhaps begs the question – how can we visualise the modelled error structure on the plot? Consider that at every single position along the gradient (x_i), there is an associated mean (μ_i) and error distribution which (typically, at least for abundance data) depends on the mean. A plot showing the quantiles for the error distribution along a (practically continuous) series of x_i values along the gradient can therefore be very helpful.

We shall show this for the *Antimora microlepis* dataset, by way of example. We start by loading the ZIM library, useful here because it has quantile functions for zero-inflated distributions.

```
library(ZIM)
```

Consider the model comprised of the mixed gaussian mean function, with zinb errors. We have estimates of the mean, μ_i, for the negative binomial in the ZINB mixture model. We already plotted these values above; they are provided as fitted values from the *senlm* model object at a chosen position (or series of positions, e.g., as provided in the variable called '`predict.x`' in the

[3] As an aside, this species is perhaps a little unusual, because most of the species in the NOAA dataset were well-modeled using zero-inflated error distributions that were linked to the mean. Nevertheless, the general take-home message here stands.

above code) along the gradient using the '`predict()`' function. Let's call these "mu" here for clarity in what follows.

```
mu = yfit.mixg.zinb
```

The estimates of the other parameters that we shall need to specify the error distribution fully are also provided within the fitted *senlm* model object.

```
theta = mixg.zinb$theta
```

We can therefore generate the quantile values for the full ZINB model for each of these positions as well.

```
# Specify the quantiles of interest.
quants <- c( seq(0.1,0.9, by = 0.1), 0.95 )

# Choose some colours.
mycols3 <- c("firebrick1","indianred1","orange1","olivedrab1","seagreen3",
             "turquoise","royalblue","slateblue","violet","violetred3")

# Create a matrix where quantiles will be held.
my.quants <- matrix(rep(NA, length(quants)*length(predict.x)),
                    ncol = length(quants), nrow = length(predict.x) )

# Get the quantiles for a ZINB distribution for every value
# of mu along the gradient.
for (ix in 1:length(predict.x) ) {
   my.quants[ix,] = qzinb(quants, k = theta["phi"], lambda = mu[ix],
                     omega = theta["pi"],
                     lower.tail = TRUE, log.p = FALSE)
   }
```

Note that we have to be careful to name each parameter in the correct way when passing these to the `qzinb()` function. The dispersion parameter (called '`phi`' in the *senlm* package) is called '`k`' in the `qzinb()` function, '`mu`' is called '`lambda`', and the zero-inflation parameter (called '`pi`' in the *senlm* package) is called '`omega`' in the `qzinb()` function.

Having achieved this, we are now able to proceed with creating the desired plot.

```
# Start with the basic scatterplot, as usual
plot(dat2$x, dat2$y, las = 1, xlab = "Depth (m)",
     ylab = "Count per trawl", ylim = c(0,80) )
points(dat2$x, dat2$y, pch = 21, col = mygrey, bg = mygrey)
lines(predict.x, mu, col = "black", lwd = 2)

# Now add the quantiles from the zinb distribution onto the plot...
for (i in 1:length(quants)) {
      lines(predict.x, my.quants[,i], col = mycols3[i], lwd = 2)
 }

# and add a legend.
legend.names <- paste(".", c(seq(10,90,by = 10), 95), sep = "")
legend(x = 0, y = 80, legend = legend.names, lwd = 2, col = mycols3)
```

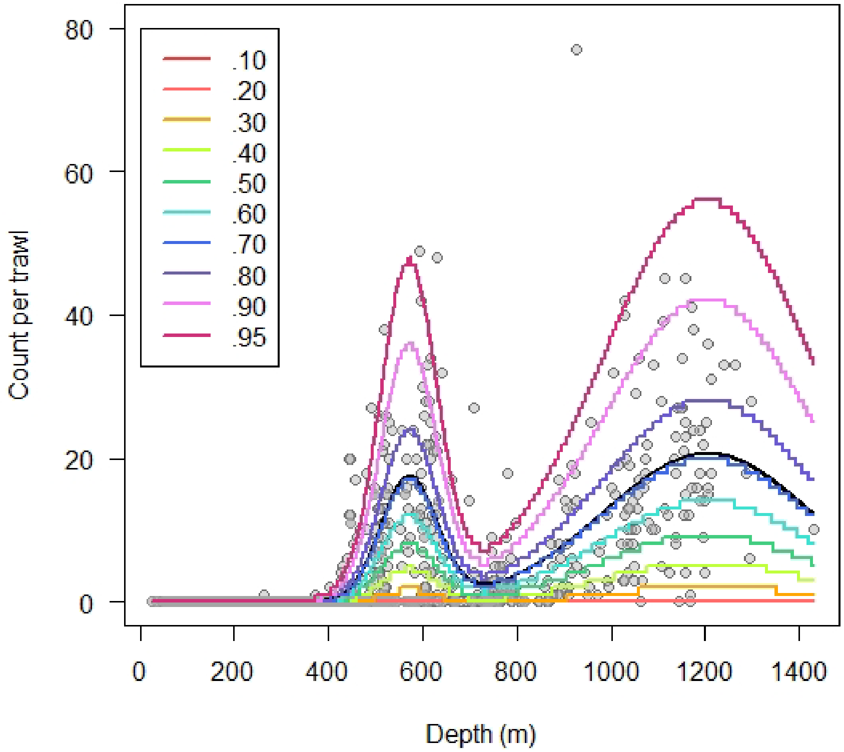

The error distribution, coupled with the mixed-Gaussian mean function, seems to have captured the overall shape of the species' response rather well in this case.
