# Supplementary material for: Non‐linear models of species' responses to environmental and spatial gradients
Source: Ecol Lett. 2022 Oct 21;25(12):2739–52. doi: 10.1111/ele.14121 (PMC9828393; doi:10.1111/ele.14121)

Fig. S1. Distributions of ΔAICc values from models fitted using each of nine different *senlm* mean functions (with the error distribution from the best AICc model) for $n = 137$ fish species.

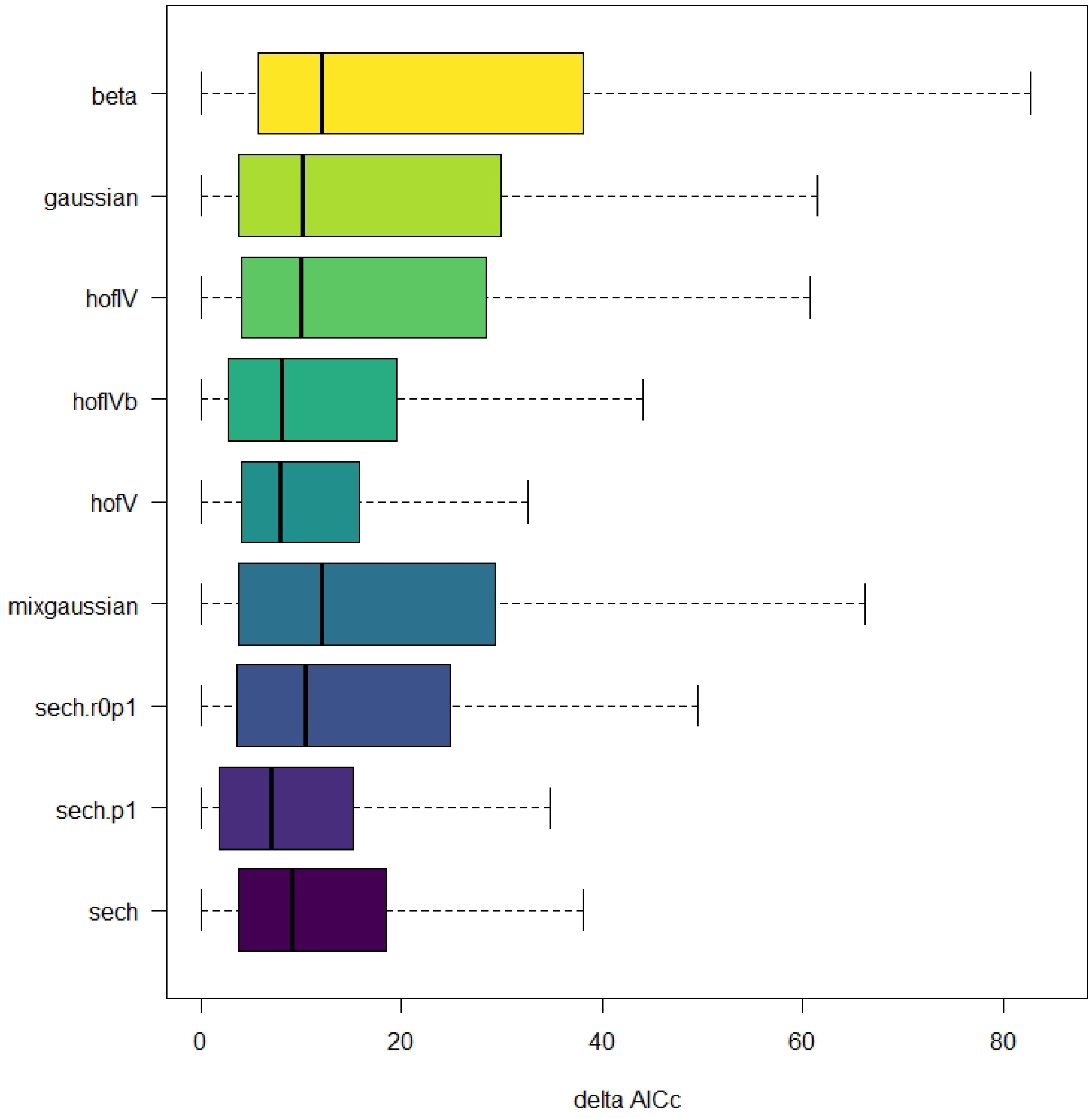

Supplement: Supplementary file 4 — Figure S1.pdf [file ELE-25-2739-s001.pdf]
