# Supplementary material for: Non‐linear models of species' responses to environmental and spatial gradients
Source: Ecol Lett. 2022 Oct 21;25(12):2739–52. doi: 10.1111/ele.14121 (PMC9828393; doi:10.1111/ele.14121)
Supplement: Supplementary file 8 — Simulation study S4.pdf [file ELE-25-2739-s006.pdf]

Simulation_study_S4.pdf, Supporting Information

A modest simulation study to compare *senlm* models with splines

Rationale

We wished to examine how well the proposed parametric *senlm* models perform by comparison with more empirical approaches, such as smooth splines and spline-based generalized additive models (GAMs[1]), at identifying the true modal position (m) and mean abundance (height, H) of a given species along a gradient. More specifically, our aim here is to use simulations to obtain empirical measures of bias and variance in the estimation of m and of H using these different methods under a range of simulated (known) scenarios. We also wished to obtain empirical measures of coverage probabilities for confidence intervals generated using standard errors of *senlm* estimates calculated using the Hessian (as opposed to using the bootstrap, jacknife, or other computer-intensive approach).

A large empirical investigation of the overall statistical properties of *senlm* models is outside the scope of the present contribution and is not our aim. Rather, we articulate a set of specific scenarios under which we would expect results obtained using spline-based models to differ from the results obtained using a formal parametric non-linear modelling approach implemented by *senlm*. It will be sufficient, for our purposes, to demonstrate a range of scenarios that are likely to be encountered with real data, and where *senlm* would provide a preferable approach to using splines for characterizing species' responses. Below we outline specific hypotheses regarding three potential factors (clearly a non-exhaustive list) that we expected would affect measures of bias and variance for splines vis-à-vis *senlm*.

1. ***Sample size.*** All methods are expected to improve in performance, generally, with increases in sample size – specifically, we expect the variance in estimates of m or H to decrease with increasing sample size (n). In addition, given that splines are more strongly empirical in nature, we expect they will rely more heavily on the individual sample points of a given dataset, so we expect they will perform more poorly (i.e., produce more variable estimates of m and H) than *senlm* for a given sample size.
2. ***Asymmetry.*** Asymmetry in a species' unimodal response along a gradient will make it more difficult for spline-based models to locate the true modal position, due to inevitable noise along the broad "tail" that would be seen in typical empirical data. In contrast, we expect *senlm* models to remain unbiased, despite asymmetry.
3. ***Zero-inflation.*** We expect that the performance of splines will be negatively affected by increases in zero-inflation. Specifically, we expect excess zeros will bias estimates of H downwards for splines, whereas *senlm* models will not be affected, provided a suitable zero-inflated error distribution is coupled with the nonlinear mean function in the *senlm* model.

---

[1] We note in passing that *senlm* models are, in fact, a type of GAM. However, for our purposes, we shall use the word "GAMs" herein to refer to the suite of spline-based approaches (B-splines, P-splines, etc.) with which this particular acronym has now generally been aligned.

Methods

Three sets of simulations were performed to investigate the above three hypotheses and to characterize empirically the performance of several core *senlm* methods and spline-based models. In each case, we generated n_{sim} = 500 datasets for all combinations of the factor(s) of interest (shown in curly brackets in what follows).

Scenario 1. Using the "haul" dataset in the R package *senlm*, we fit a model with a Gaussian unimodal mean function coupled with negative binomial (NB) errors to abundance values for the fish species *Sebastolobus altivelis* versus the depth gradient, which yielded the following parameter estimates: $\theta_M = \{H = 1548.3177; m = 904.3537; s = 159.928\}$ and $\theta_E = \{\phi = 2.4906\}$. Asserting these estimates to be "true" parameters for this scenario, we generated $n_{sim} = 500$ simulated datasets using the Gaussian mean model + NB errors having those parameters for each of the following sample sizes: $n = \{10, 20, 50, 100, 250, 500, 750, 1000\}$. Specifically, for a given sample size n, a sequence of $i = 1, \ldots, n$ equally spaced values of x_i along the depth gradient were generated between 20 m and 1600 m. At each of these positions, x_i, a corresponding value of y_i was drawn randomly from $NB(\mu_i, \phi)$, where the mean value was calculated using the Gaussian nonlinear function $\mu_i = f(x_i; \theta_M)$ with known "true" parameters θ_M. Each simulated dataset was then analysed using the following methods, with the core aim being to estimate m and H:

(i) Gaussian + NB (*senlm* model with: `mean_fun="gaussian", err_dist="negbin"`)

(ii) sech + NB (*senlm* model with: `mean_fun="sech", err_dist="negbin"`)

(iii) B-spline (function `smooth.spline()` in R), with estimates of m and H derived empirically from the resulting fitted curve.

(iv) P-spline + NB (obtained using R package *mgcv* and the following code: `gam( y ~ s(x, bs = "ps"),… optimizer = "outer", family = nb() )`), with estimates of m and H derived empirically from the resulting fitted curve.

We plotted the distribution of values (of either m or H) estimated using each of the above four methods (colours) for the 500 simulated datasets *vs* sample size (n), using boxplots, to visually compare characteristics of the methods in terms of relative bias and variance.

Scenario 2. The NOAA trawl dataset described in the main text (see "Example" in the main text) includes count data for the fish species *Sebastolobus alascanus*, which was clearly asymmetric in its distribution along the depth gradient (Fig. 1). To create values for θ_M and θ_E, we fit an *senlm* model to these data with: `mean_fun="sech.p1", err_dist="negbin"`. The resulting parameter estimates were then used as the "true" parameters for the ensuing simulations to exemplify an asymmetric case, as follows: $\theta_M = \{H = 80.9096; m = 295.8135; s = 36.3725; r = 0.86756; p = 1\}$ and $\theta_E = \{\phi = 1.8475\}$. Note that the positive value of r under the sech model indicates a mean functional curve with a modal position shifted towards the left and a rather large right-hand tail (Fig. 2). The simulations for this scenario were done in the same way as described for Scenario 1, except that data were generated using a sech mean model + NB errors and with different "true" parameters, θ_M and θ_E,as indicated. We used the same four methods outlined above to analyse each simulated dataset, and summaries and comparisons of these methods' relative performance were

achieved in the same manner as described above. Coverage probabilities were also estimated under this scenario, as described above.

Scenario 3. To examine the additional effects of zero-inflation on model performance, we generated data as described under Scenario 2 using a sech mean function, but with ZINB errors. Specifically, all parameters in θ_M and also the value of ϕ remained as described under Scenario 2, but values of y_i were drawn from $ZINB(\mu_i, \phi, \pi)$. For each of the following sample sizes: $n = \{10, 20, 50, 100, 250, 500\}$, we generated 500 simulated datasets for each of the following values of $\pi = \{0.01, 0.10, 0.20, 0.40, 0.60, 0.80\}$. Recall that π is the parameter corresponding to the proportion of excess zeros in the model. For each simulated dataset, the same 4 methods described above were used to estimate H and m. We note in passing that we were not able to find a spline-based method that could handle ZINB errors explicitly in a robust fashion under simulation[2]. Summaries, comparisons and coverage probabilities were assessed precisely as previously described for Scenarios 1 and 2.

Coverage. For all *senlm* models that were used to estimate parameters under the above three scenarios, we calculated a 95% confidence interval for H and for m (using the Hessian matrix) for each simulated dataset. We estimated coverage probability, in each case, as the proportion of intervals (out of 500) that contained the (in this case known) true values of H and m, respectively. The normal approximation of a confidence interval for the binomial is: $p \pm z_{1-\alpha/2}\sqrt{p(1-p)/n_{sim}}$. Here, we have $p = 0.95$ and $n_{sim} = 500$; thus, for $\alpha = 0.05$, $z_{0.95} = 1.96$, and so we expect methods performing well generally should yield an empirical coverage that lies in the interval $\{0.9309, 0.9691\}$.

Results

Scenario 1. When data were generated from a **symmetric** (Gaussian) mean response curve with NB errors, both the parametric (Gaussian or sech) and the non-parametric (P-spline or B-spline) approaches estimated m without any clear bias, and all methods also improved in precision with increases in sample size (Fig. S4.1a). Three methods (Gaussian+NB, sech+NB and P-spline+NB) also showed no bias in the estimation of H, but the B-spline approach clearly under-estimated H for small to moderate sample sizes ($n \leq 500$; Fig. S4.1b). However, the variance in the estimators (spread) was greater for the spline methods than for the parametric nonlinear modelling approaches, even for very large sample sizes ($n \geq 500$). In addition, performance was slightly better for the Gaussian than for the sech model (i.e., it had lower variance in the estimators at a given sample size), which was not surprising, given that simulations were produced using a Gaussian mean response curve (Fig. S4.1).

Scenario 2. When data were generated from an **asymmetric** mean response curve with NB errors, the sech model clearly out-performed all other modelling approaches (Fig. S4.2). Although we expected the sech+NB model to have the best performance (given that this was the model used to generate the data), this does not detract from the fact that the other

[2] We note that the '*VGAM*' package for R offers the option to include ZINB errors, but in practice, we found there were a large number of data-specific convergence problems, even for very large datasets. This may have been caused by the dual challenge of simultaneously estimating both the dispersion parameter (ϕ) and the zero-inflation parameter (π) of the ZINB in the context of a flexible GAM. This issue warrants further study; for our purposes, it rendered the VGAM+ZINB approach unfeasible to include in this simulation study.

modelling approaches produced biased estimates. More specifically, given that the true modal position was shifted towards the left and there was a rather large right-hand tail in the true mean response, the Gaussian model and the spline models all consistently overestimated the value of m, even for very large sample sizes (Fig. S4.2a). They also showed biases in the estimation of H: (i) P-splines tended to underestimate H for small sample sizes ($n \leq 250$), and to overestimate them for large sample sizes ($n \geq 500$); (ii) B-splines tended to underestimate H, although this did improve somewhat for larger sample sizes ($n \leq 500$); and (iii) Gaussian models always dramatically underestimated H (Fig. S4.2b). We note in passing that the specific direction of these biases would clearly be altered by a change in the direction of the asymmetry. Another feature to note is that none of the models performed particularly well for this asymmetric scenario (i.e., all had high variance and low accuracy) if sample sizes were small ($n \leq 100$; Fig. S4.2).

Scenario 3. When zero-inflation was included in an asymmetric data-generating model, the methods all showed some decreases in their overall performance with increases in the proportion of excess zeros (Fig. S4.3). Lack of desirable performance was most apparent for the spline models: both the bias and the variance of estimated values of either m or H increased markedly for P-spline and B-spline models as zero-inflation increased. The Gauss+ZINB approach, despite its explicit inclusion of a zero-inflated error structure, also yielded highly variable and imprecise results for these scenarios, presumably caused largely by asymmetry in the mean response. The best results were obtained using either the sech+ZINB or the sech.p1+ZINB parametric modelling approaches, with the latter approach (having one fewer parameter than the more general sech model, because it includes the constraint of $p = 1$) showing no clear bias and only modest increases in variance even when the degree of zero-inflation was massive (i.e., when $\pi = 0.8$, corresponding to an 80% chance of sampling an excess zero in simulated data).

Coverage. For symmetric (Gaussian + NB) scenarios, empirical coverage of CIs derived from variances in parameters estimated using the Hessian matrix under maximum likelihood was anti-conservative for small sample sizes ($n \leq 20$, Fig. S4.4). Empirical coverage in estimates of either m or H readily converged to the nominal level (95%) for moderate to large sample sizes ($n \geq 50$ for symmetric cases and $n \geq 100$ for asymmetric cases; Fig. S4.4).

Conclusions

The parametric *senlm* models outperformed the more flexible spline-based approaches for the scenarios investigated here. We consider that spline-based approaches, although clearly more flexible than specific parametric models, are also more strongly affected by idiosyncrasies of individual datasets. Specifically, in symmetric cases, estimates of the modal position of a species along a gradient (m) derived from spline-based models consistently had higher variance than parametric nonlinear models (Gaussian or sech), even for large sample sizes ($n \geq 500$). Spline-based models also demonstrated a clear bias in the estimation of either the modal position (m) or the mean abundance of the species (height, H) at that modal position, under scenarios where there was asymmetry in the mean response. Furthermore, both the bias and the variance of estimators worsened for spline-based approaches with increases in the degree of zero-inflation. However, both zero-inflation and asymmetric responses of species

along environmental gradients are truly commonplace in the vast majority of broad-scale ecological studies (e.g., see Table 2 in the main text). A further advantage of the parametric models is that parameter estimates are obtained directly, rather than indirectly. Variances in parameters can also be estimated directly using the Hessian matrix. For moderate-to-large sample sizes ($\sim n > 50$), these can be used to build confidence intervals with correct coverage at a chosen level (e.g., 95%).

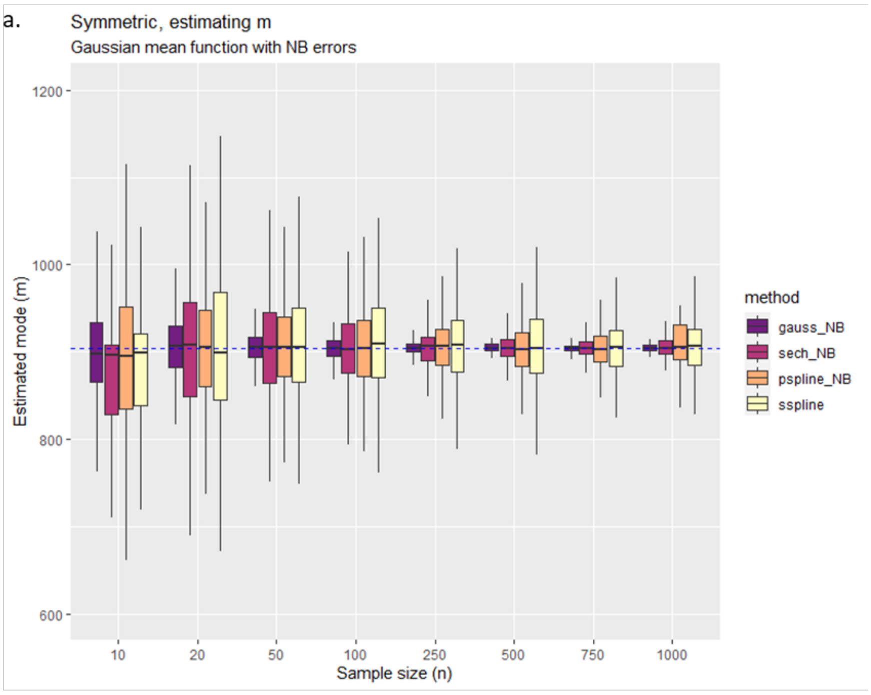

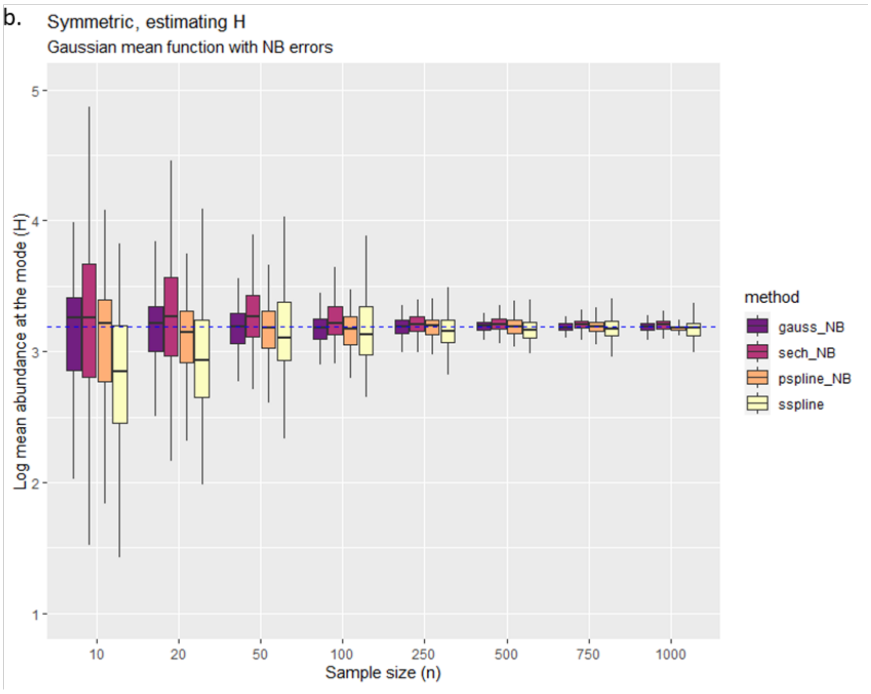

Fig. S4.1. Box-and-whisker plot of values for (a) m, modal position, and (b) H, mean abundance at the modal position, *vs* a range of sample sizes (n), estimated using each of 4 different methods (colours) for $n_{sim} = 500$ datasets generated from a symmetric species-environment model (referred to in the methods as "Scenario 1", having Gaussian mean function and negative binomial errors). Outlying points (beyond the whiskers) have been omitted for clarity.

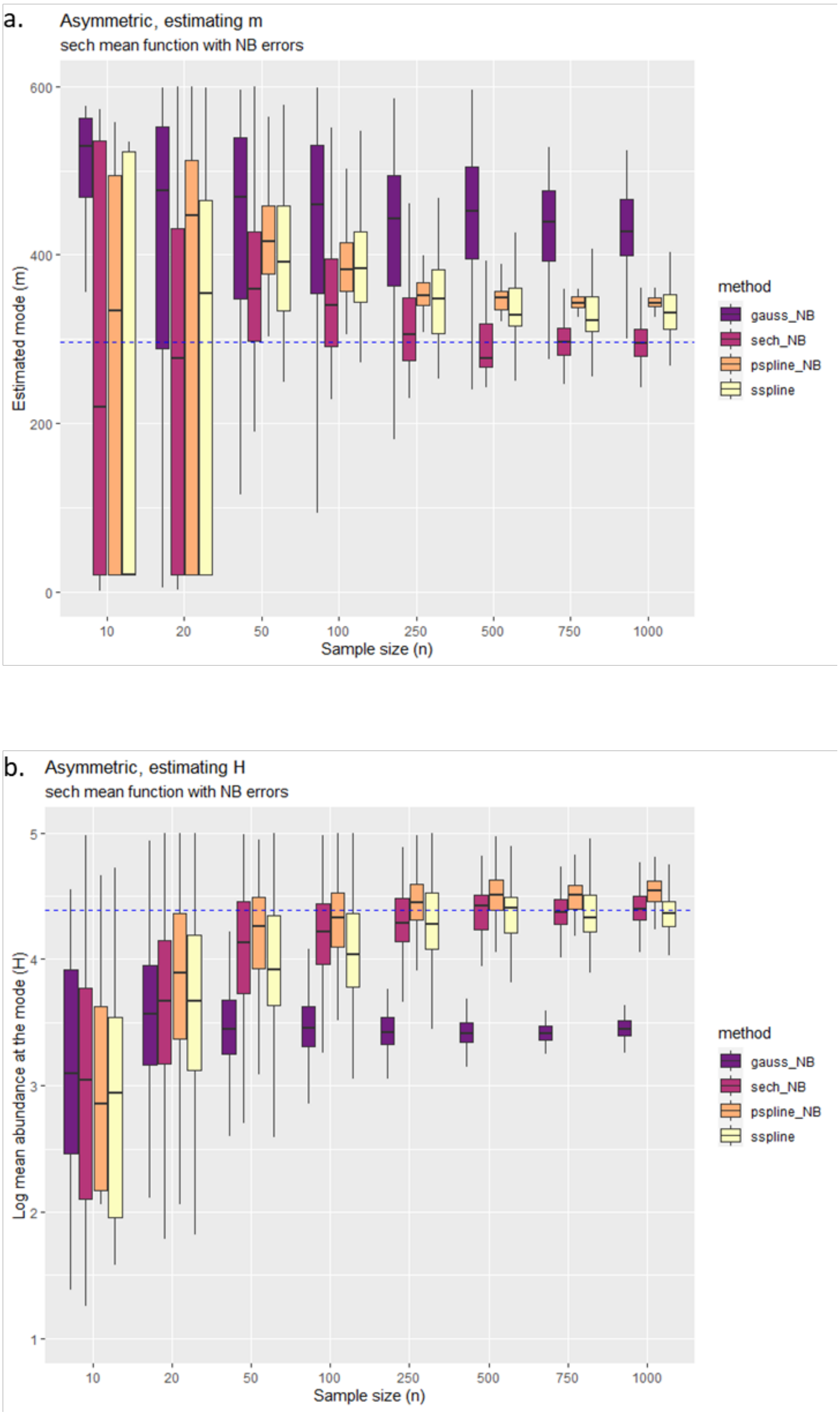

Fig. S4.2. Box-and-whisker plot of values for (a) m, modal position, and (b) H, mean abundance at the modal position, *vs* sample size (n), estimated using each of 4 different methods (colours) for $n_{sim} = 500$ datasets generated from an asymmetric species-environment model (referred to in the methods as "Scenario 2", having a sech mean function and negative binomial errors). Outlying points (beyond the whiskers) have been omitted for clarity.

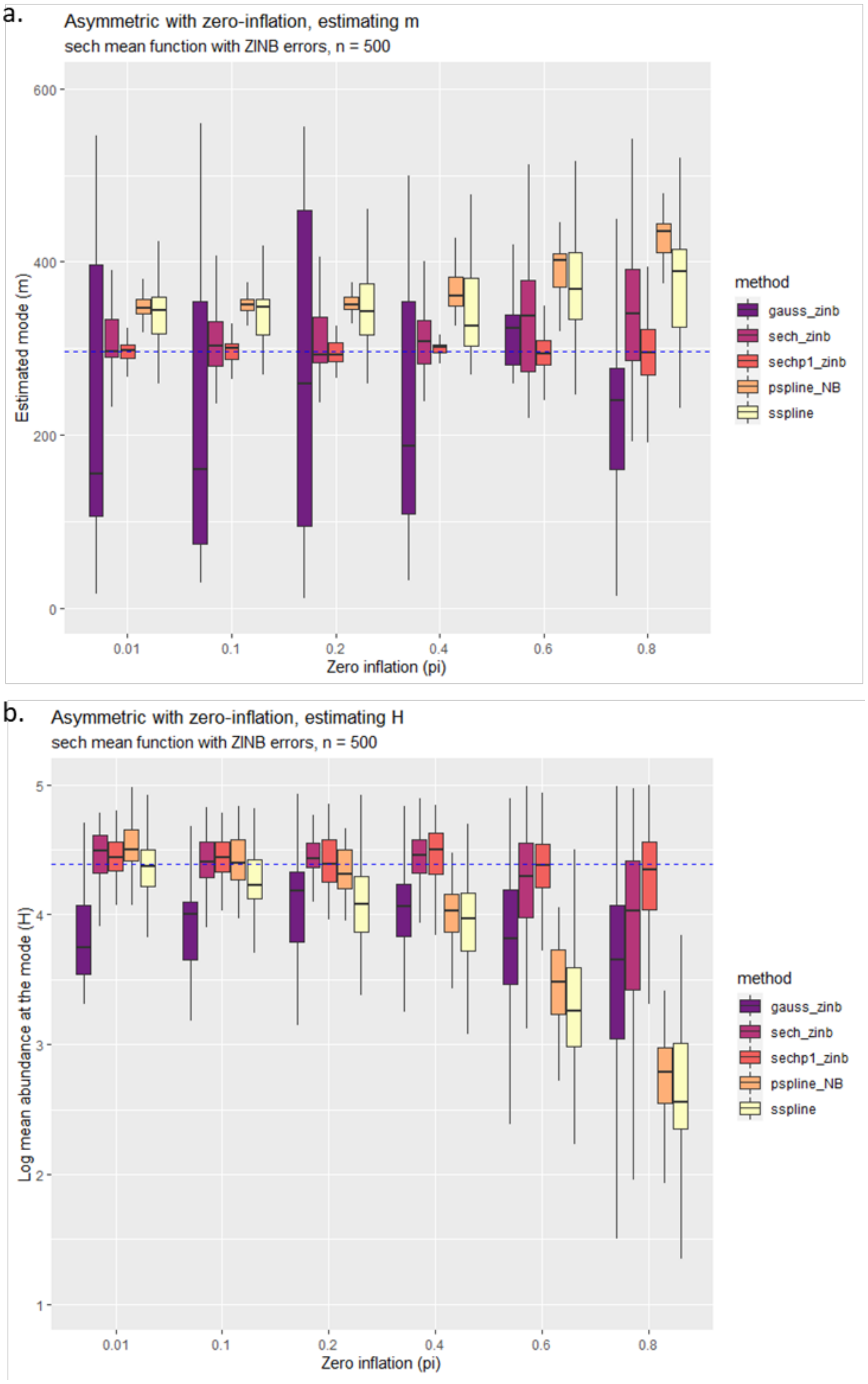

Fig. S4.3. Box-and-whisker plot of values for (a) m, modal position, and (b) H, mean abundance at the modal position, *vs* degree of zero-inflation (π), estimated using each of 5 different methods (colours) for $n_{sim} = 500$ datasets, each having a sample size of $n = 500$, generated from an asymmetric species-environment model with zero-inflation (referred to in the methods as "Scenario 3", having a sech mean function and zero-inflated negative binomial errors). Outlying points (beyond the whiskers) have been omitted for clarity.

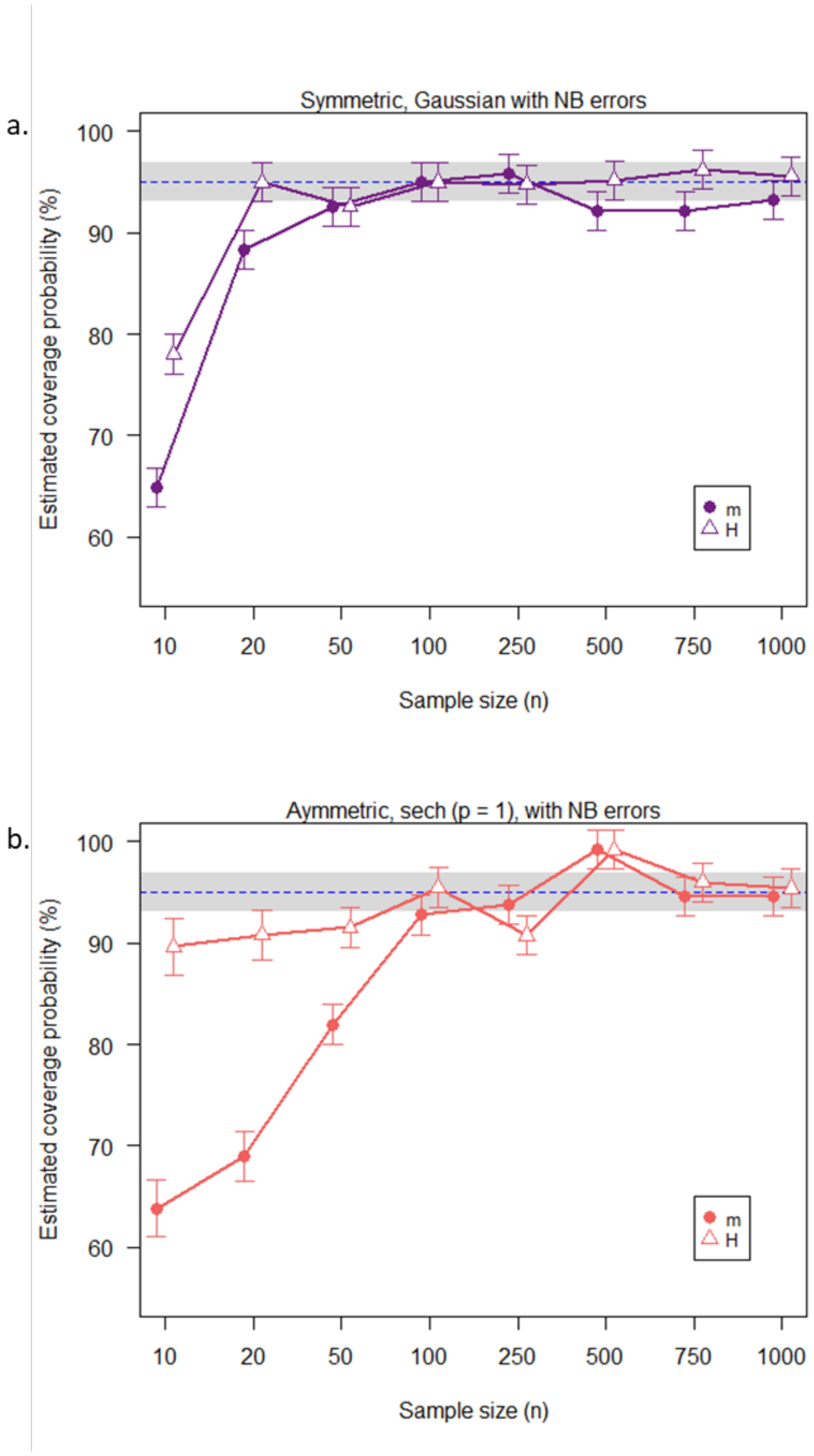

Fig. S4.4. Empirical coverage probability (%) of confidence intervals (CIs) for either m or H (symbols) built using the Hessian matrix from *senlm* models *vs* a range of sample sizes (n), for $n_{sim} = 500$ datasets generated from either: (a) the symmetric Gaussian model with NB errors (Scenario 1) or (b) the asymmetric sech model with NB errors (Scenario 2). The dotted blue line is the *a priori* chosen level (i.e., 95%). Given $n_{sim} = 500$ trials, the 95% confidence interval for the empirical coverage (i.e., {93.09%, 96.91%}) is shown as a grey horizontal strip.
